# Inherent single-cell heterogeneity of the transcriptional response to hypoxia in cancer cells

**DOI:** 10.1093/narcan/zcaf021

**Published:** 2025-08-28

**Authors:** Małgorzata Wilk, Thomas Knöpfel, Stana M Burger, Stellor Nlandu Khodo, Roland H Wenger

**Affiliations:** Institute of Physiology, University of Zürich, Zürich, CH-8057, Switzerland; Institute of Physiology, University of Zürich, Zürich, CH-8057, Switzerland; Institute of Physiology, University of Zürich, Zürich, CH-8057, Switzerland; Institute of Physiology, University of Zürich, Zürich, CH-8057, Switzerland; Institute of Physiology, University of Zürich, Zürich, CH-8057, Switzerland

## Abstract

Hypoxia-inducible factor (HIF) is a master regulator of cancer cell adaptation to tumor hypoxia and is involved in cancer progression. Single-cell (sc) differences in the HIF response allow for tumor evolution and cause therapy resistance. These sc-differences are usually ascribed to tumor microenvironmental differences and/or clonal (epi)genetic variability. However, the sc-heterogeneity of the HIF response in otherwise identical cells cultured under defined *in vitro* conditions has not yet been addressed. Therefore, we analyzed the sc-response to hypoxia in nonclonal cell lines and multiple clonal derivatives, including HIF-1α or HIF-2α knockouts. While HIF-1α and HIF-1 target mRNA sc-heterogeneity was slightly higher than global transcription or specific housekeeping messenger RNAs (mRNAs), HIF-2α and especially HIF-2 target mRNA sc-heterogeneity was extraordinary, and remained in independent clones following HIFα knockouts. Unexpectedly, neither HIF-2α mRNA nor nuclear protein levels correlated with target mRNA levels. Unsupervised but not supervised HIF target gene dimensionality reduction revealed the initial sample composition after scRNA-seq, demonstrating that, owing to sc-heterogeneity, individual HIF target genes are not sufficient to unequivocally identify hypoxic cancer cells. In conclusion, the pronounced intrinsic sc-heterogeneity of the HIF response represents a hitherto unrecognized feature of cancer cells that impairs clinical HIF pathway-dependent cancer cell identification and targeting.

## Graphical Abstract

**Graphical Abstract:**
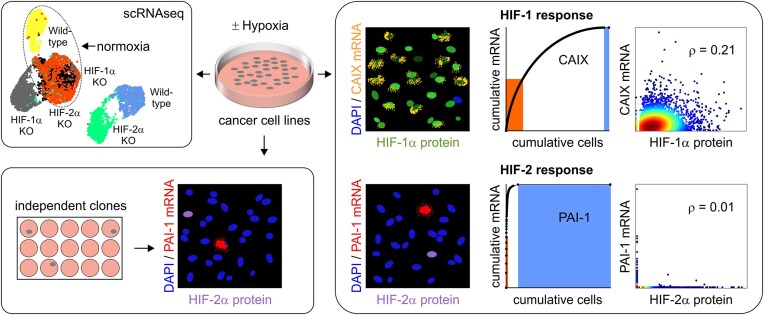

## Introduction

During the growth of a solid tumor, irregular tissue architecture and (epi)genetic instability cause cancer cell heterogeneity at the single-cell (sc) level. The resulting pool of distinct cancer cells is under constant microenvironmental evolutionary selection pressure, representing a major mechanism of tumor progression, metastasis, immune evasion, and eventually treatment escape. Tumor sc-heterogeneity is largely based on extrinsic microenvironmental and intrinsic (epi)genetic factors. Owing to their rapid proliferation and high metabolic demand, combined with disrupted microvessel organization and inflammation, oxygen consumption by tumor cells often exceeds the oxygen supply. In turn, subsequent regional tumor hypoxia reprograms intrinsic processes in cancer cells.

Hypoxia-inducible factor (HIF) is a master regulator of cellular adaptation to hypoxia. HIF is a heterodimeric transcription factor consisting of one of three oxygen-labile α-subunits and a common constitutive β-subunit. HIF-mediated transcriptional responses have been shown to support the ability of tumor cells to cope with tissue hypoxia and cancer therapy [1, 2]. It is well-established that the HIF response contributes to cancer stem cell maintenance, metabolic adaptation, (epi)genetic instability, angiogenesis, metastasis, immune evasion, and treatment resistance [3–7]. Because tumor angiogenesis and perfusion are highly irregular and dynamic processes, tumor microenvironmental hypoxia causes spatial and temporal variability in the HIF-mediated transcriptional response [8, 9]. While this extrinsic sc-variability of the HIF response is generally well recognized, the potential intrinsic sc-heterogeneity of the HIF response in otherwise largely identical clonal cell pools has not been considered thus far. We therefore used monoclonal cancer cell lines under relatively homogenous *in vitro* culture conditions to address this crucial question.

Because the transcriptional HIF response is mediated mainly by HIF-1 and HIF-2, we also addressed whether variable expression levels of the two oxygen-labile HIFα isoforms contribute to the sc-heterogeneity of cancer cells. While the *HIF1A* gene (encoding HIF-1α) is ubiquitously expressed, the *EPAS1* gene (encoding HIF-2α) shows a more restricted expression pattern *in vivo*. However, at least in batch analyses of dedifferentiated cancer cells, these two HIFα isoforms are often coexpressed, but whether they functionally overlap within the same cell at the same time remains to be investigated.

## Materials and methods

### Cell culture

MCF-7 triple-positive breast adenocarcinoma, MDA-MB-231 triple-negative breast adenocarcinoma, Hep3B2.1–7 hepatocellular carcinoma (Hep3B), and U2OS osteosarcoma cell lines were obtained from American Type Culture Collection (ATCC; Manassas, VA, USA). All cell lines were authenticated via highly polymorphic short tandem repeat loci (September to November 2023; Microsynth, Balgach, Switzerland). All the cells were cultured in high-glucose Dulbecco’s modified Eagle’s medium (DMEM; Sigma–Aldrich, Burlington, MA, USA) in humidified air containing 5% CO_2_ at 37°C, resulting in 18.5% O_2_ normoxic conditions [10]. The media were supplemented with 10% heat-inactivated fetal bovine serum (Thermo Fisher Scientific, Waltham, MA, USA), 100 μg/ml streptomycin, and 100 U/ml penicillin (Sigma–Aldrich). Before seeding, all cells were filtered through a cell strainer (pluriStrainer 30 μm; pluriSelect, Leipzig, Germany). HIF was induced by hypoxia (0.2% O_2_) in an InvivO_2_ 400 workstation (Baker Ruskinn, Bridgend, UK) or by treatment with 100 μM FG-4592 (roxadustat; Selleckchem, Houston, TX, USA). Cell density at harvesting did not exceed 50%, and cell viability was >92% under normoxia and >86% after 48 h of hypoxia, as determined by trypan blue exclusion (Vi-Cell XR; Beckman Coulter, Brea, CA, USA).

### Generation of HIFα knockdown and knockout cell lines

Stable HIF-1α and HIF-2α knockdown by RNA interference in MCF-7 cells was described previously [11]. The HIF-1α and HIF-2α knockout (KO) cell lines were generated via CRISPR–Cas9 gene editing technology, using the manufacturer’s own batch of MCF-7 cells (Synthego Corporation, Redwood City, CA, USA). Single guide RNAs (sgRNAs) targeting exon 2 of the *HIF1A* (5′-CCUCACACGCAAAUAGCUGA-3′) or *EPAS1* (5′-AGAAGUCCCGGGAUGCUGCG-3′) genes, respectively, or a nontarget control (NTC) sgRNA were used. Monoclonal KO and NTC cell lines were obtained via limited dilution cloning. Biallelic gene disruption was confirmed by genomic polymerase chain reaction (PCR) (primers are listed in Supplementary Table S1) and deep sequencing (Microsynth). The sequencing results are shown in Supplementary Fig. S1A and B. Immunoblotting confirmed the KOs (Supplementary Fig. S1C and D).

### RNA quantification by RT-qPCR

Total RNA was extracted as described previously [12]. Complementary DNA (cDNA) was generated via reverse transcription (RT) of 2 μg of total RNA using AffinityScript reverse transcriptase (Agilent Technologies, Basel, Switzerland). Quantitative (q) PCR was performed in an AriaMx real-time PCR system (Agilent Technologies) via a SYBR Green qPCR reagent kit (Sigma–Aldrich). The primers used were purchased from Microsynth and are listed in Supplementary Table S1. Calibrated standards were included for copy number determination. All the messenger RNA (mRNA) data are expressed as ratios relative to the mRNA levels of the housekeeping ribosomal protein L28.

### 5-ethynyluridine incorporation

To assay global RNA transcription, cells were seeded on Nunc Lab-Tek II 8-well chamber slides (Thermo Fisher Scientific), and 100 μM 5-ethynyluridine (5-EU; Baseclick, Munich, Germany) was added 30 min before fixation with 4% paraformaldehyde (PFA; Sigma–Aldrich) for 20 min and permeabilization with 0.5% Triton X-100 (Sigma–Aldrich) in 20 mM glycine (Biosolve Chimie, Dieuze, France) for 10 min. For the hypoxia experiments, the entire procedure was carried out in a hypoxia workstation. Ethynyl-RNA was detected by incubating the cells for 30 min in the dark with a CLICK reaction mixture consisting of 2 mM CuSO_4_, 100 mM sodium ascorbate, and 10 μM Alexa Fluor 647 azide (Thermo Fisher Scientific). Nuclei were counterstained with 1 μg/ml 4′,6-diamidino-2-phenylindole (DAPI; Sigma–Aldrich) for 20 min. The slides were mounted in Mowiol/DABCO (Millipore and Sigma–Aldrich, respectively) and imaged on an Axioscan 7 slide scanner (Zeiss Microscopy, Oberkochen, Germany).

### mRNA-fluorescence *in situ* hybridization

For fluorescence *in situ* hybridization (FISH) via RNAscope Multiplex Fluorescent v2 assays, cells were seeded as above and pretreated according to the manufacturer’s recommendations (Advanced Cell Diagnostics, Hayward, CA, USA). Following incubation for 30 min in the dark with 2 μg/ml HCS CellMask Green Stain (H322714A; Thermo Fisher Scientific), hybridization to the RNAscope probes (Supplementary Table S2) was performed for 2 h at 40°C in a HybEZ oven (Advanced Cell Diagnostics), followed by signal amplification and signal detection using Opal 570 or Opal 650 fluorescent reagents (Akoya Biosciences, Marlborough, MA, USA). Following counterstaining with DAPI, the slides were mounted with ProLong Gold antifade (Thermo Fisher Scientific) and imaged as described earlier.

### Immunofluorescence

Cells were seeded as described earlier, fixed with 4% PFA for 20 min, and permeabilized with 0.5% Triton X-100 in 20 mM glycine for 10 min. For the hypoxia experiments, the entire procedure was carried out in a hypoxia workstation. To prevent nonspecific antibody binding, the cells were incubated with 5% bovine serum albumin for 30 min. Primary antibodies (Supplementary Table S3) were allowed to bind overnight at 4°C. Following incubation with secondary antibodies for 30 min in the dark at 37°C, the cells were counterstained with 1 μg/ml DAPI for 30 min at 37°C, mounted in Mowiol/DABCO, and imaged as described earlier. For sequential immunofluorescence (IF), the Opal 4-color kit was used according to the manufacturer’s recommendations (Akoya Biosciences), and the cells were prepared as earlier but fixed with 10% neutral buffered formalin (NBF; Sigma-Aldrich) for 30 min.

### Image quantification

Following manual elimination of low-quality images, image quantification was performed via CellProfiler version 4.2 (CellProfiler software, Cambridge, MA, USA). Negative control samples were used to define the minimal detection thresholds. Cell borders were defined with the use of HCS CellMask Green Stain (Thermo Fisher Scientific). 5-EU, mRNA, or protein signals (“primary objects”) were related to “cell objects” identified with DAPI and HCS CellMask stainings. The background was subtracted through the “MaskObjects” module. The integrated intensity (total intensity) module was used to define per-cell 5-EU, mRNA, or protein levels. Typically, 10 000–20 000 cells were evaluated for the determination of a heterogeneity index. To compare different Opal dyes of variable fluorescence brightness, the intensities of the genes of interest were normalized to the mean value of the cellular intensities over all cells of a housekeeping POLR2A or UBC RNAscope probe labeled with the corresponding Opal dye.

### Immunoblotting

Cells were washed with phosphate-buffered saline (PBS), and the proteins were extracted for 15 min on ice with 10 mM Tris–HCl (pH 8.0), 1 mM ethylenediaminetetraacetic acid (EDTA), 400 mM NaCl, 0.1% Nonidet *P*-40, freshly supplemented with protease inhibitor cocktail, 1 mM NaF, and 10 mM Na_3_VO_4_ (all obtained from Sigma–Aldrich). The protein concentration was estimated via the bicinchoninic acid (BCA) method (Thermo Fischer Scientific), and 40–80 μg of total protein was separated by sodium dodecyl sulphate–polyacrylamide gel electrophoresis, followed by electrotransfer to nitrocellulose membranes (Protran Premium 0.2 μm; Amersham, Cytiva, Marlborough, MA, USA). Primary antibodies (Supplementary Table S4) were allowed to bind overnight at 4°C, followed by incubation with secondary antibodies for 1 h at room temperature. For the detection of horseradish peroxidase activity, SuperSignal enhanced chemiluminescence substrate (Thermo Fischer Scientific) was added, and the signal was recorded with a Fusion FX7 camera (Vilber, Eberhardzell, Germany).

### scRNA sequencing

MCF-7 cells were incubated under normoxic or hypoxic conditions for 48 h and harvested with 0.5 mg/ml porcine trypsin in 0.5 mM EDTA (pH 8.0) (Sigma–Aldrich). A total of 20 000 cells were distributed under the same oxygen conditions on HIVE Collectors (Honeycomb Biotechnologies, Waltham, MA, USA). The cells were treated with Sample Preservation Solution, followed by the addition of Lysis Solution and library preparation according to the manufacturer’s instructions (Honeycomb Biotechnologies). Library quality was verified with high-sensitivity D5000 ScreenTape (Agilent Technologies). Average cDNA lengths were 1807, 1770, and 1660 bp for the three libraries MCF-7 NTC, MCF-7 HIF-1α KO, and MCF-7 HIF-2α KO, respectively (normoxic and hypoxic samples combined). For scRNA-seq, custom sequencing primers from Honeycomb were used with a NextSeq500 instrument (Illumina, San Diego, CA, USA). On average, 1093 cells were sequenced per sample, with an average of 147 606 reads per cell (read length: 25 for barcode/UMI plus 50 for RNA). The human reference genome (GRCh38.p13 with gene model definition GENCODE release 37) was used in the R package ezRun (https://doi.org/10.5281/zenodo.10101864) and subsequently filtered for protein-coding, ribosomal RNA (rRNA), transfer RNA, and mitochondrial RNA gene biotypes [13]. Sample read alignment against the aforementioned genome and feature-barcode count matrix generation was performed with BeeNet v1.1.2 pipeline (Honeycomb Biotechnologies), using 5000 barcodes per sample. Downstream analysis was performed on the feature-barcode count matrices using the R package Seurat v4.2.1 [14]. Cells were further filtered according to hard thresholds for very low RNA counts (<1000 TPM). In addition, cells were filtered according to RNA count, feature count, and mitochondrial percentage using a median-absolute-deviation (MAD) approach (nmad = 3) implemented in the package scater [15]. Cells with >0.7 riboprotein content and those marked as doublets by scDblFinder [16] were also removed. The average RNA count per cell was 8728 transcripts per kilobase million (TPM), and the average number of genes detected was 2890. Integration across all six samples was performed using the CCA approach implemented in Seurat v4.2.1 using the top 15 principal components (PCs). Filtered data were log-normalized and scaled using scTransform implemented in Seurat [17], using the 3000 most strongly expressed genes. Both supervised (using a list of the most highly induced 70 HIF target genes) and unsupervised PC analyses (PCA) were conducted on the SCTransform-normalized data. Subsequently, cell neighbors were identified using the FindNeighbors function applied to the PCA embeddings. Clustering was performed with FindClusters at a low resolution (0.1) to detect fine-grained cell subpopulations [18]. Uniform manifold approximation and projection (UMAP) was applied to visualize the cellular landscape in reduced dimensions using RunUMAP based on the PCA results (https://arxiv.org/abs/1802.03426). UMAP embeddings for both supervised and unsupervised analyses were generated using the first 19 PCs. Dimensional plots (DimPlots) were colored by sample identity. To ensure consistent comparison, axis limits were standardized across plots. Differential expression analysis was conducted on scRNA-seq count data using the DESeq2 package [19]. Raw gene counts were first filtered to exclude lowly expressed genes, retaining only those with a total count >50 across all samples. Samples were grouped according to experimental conditions (normoxia or hypoxia). Differentially expressed genes (DEGs) were identified based on a log_2_(fold change) > 2 and a base mean expression > 350 TPM. Significant DEGs were then annotated by converting ENSEMBL IDs to gene symbols via the org.Hs.eg.db annotation database (https://bioconductor.org/packages/org.Hs.eg.db). For supervised clustering, a DEG list was used based on log_2_(fold change) > 1.5 and a base mean expression > 50 TPM. Normalized counts for significant DEGs were extracted, log-transformed, and *Z*-score scaled per gene to highlight expression patterns across samples. Heat maps were generated using the ComplexHeatmap package with clustering applied to genes but not samples, maintaining a biologically relevant sample order [20]. To investigate the expression of specific genes, subsets of the integrated Seurat object were created for the 3 cell lines. Feature plots of gene expression were generated on UMAP embeddings with standardized axis limits and aspect ratios to allow direct comparison between conditions.

### Tumor database analyses

Scatter plots with pairwise mRNA correlations of breast cancer data deposited in The Cancer Genome Atlas (TCGA) database (www.cancer.gov/tcga) were generated via Gene Expression Profiling Interactive Analysis (GEPIA; gepia.cancer-pku.cn) [21].

### Statistics

To analyze statistical significance, a two-tailed unpaired Student’s *t*-test or two-way ANOVA with Bonferroni’s multiple comparisons test was conducted for comparisons between two groups or three or more groups, respectively (GraphPad Prism version 10.0; GraphPad Software, San Diego, CA, USA). All results are presented as the means ± standard deviations (SDs) of the indicated number of independent experiments. *P* values < .05 were considered significant. Spearman’s *ρ* was used to assess the strength of association between two variables using Python software version 3.12.0 (www.python.org; Python Software Foundation, Wilmington, DE, USA).

## Results

### Definition of representative HIF isoform-specific target genes in MCF-7 cells

To analyze the sc-heterogeneity of the HIF response, we chose the widely used triple-positive breast adenocarcinoma MCF-7 cell line as an *in vitro* model. We and others previously reported coexpression of HIF-1α and HIF-2α as well as differential HIF-1 and HIF-2 responses in this cell line, including analyses by HIF-1/2α ChIP-seq [11, 22–25]. However, these data were generated in batch experiments and do not provide information about the HIF response at the sc-level. Therefore, we first validated the specific regulation of two prototypical HIF-1 and HIF-2 target genes, CAIX and PAI-1, respectively [11]. We initially used our in-house nonclonal MCF-7 subline stably transfected with short hairpin (sh)RNA to knock down the two HIFα isoforms and exposed them to hypoxia (0.2% O_2_) for 8–72 h. Compared with those in control (shCtrl) cells, the levels of both CAIX and PAI-1 mRNAs (Supplementary Fig. S2A) and proteins (Supplementary Fig. S2B) strongly decreased in the corresponding shHIF-1α and shHIF-2α cells. Because of the known differences in HIF-1α and HIF-2α stabilization/degradation kinetics [11, 26], we analyzed the expression kinetics of the two HIFα isoforms (Supplementary Fig. S2C). The 48 h time point seemed most suitable for the simultaneous analysis of both pathways and was hence used in all subsequent experiments, if not indicated otherwise.

HIFα isoform dependency was further confirmed in newly generated clonal HIF-1α KO and HIF-2α KO MCF-7 cell lines (Supplementary Fig. S1), which were derived from the company’s MCF-7 subline that is at least two decades distant from our in-house subline. As shown in Supplementary Fig. S2D, the mRNA levels of CAIX and PAI-1 in the analyzed KO clones were strongly induced by hypoxia and highly restricted to the transcriptional activity of HIF-1 and HIF-2, respectively. The mRNA levels of the housekeeping ribosomal protein L28 were affected neither by hypoxia nor by the HIFα KOs. Compared with the MCF-7 NTC cell line, which served as a wild-type (WT)-like control clone, the hypoxic induction of the HIF-2 target gene PAI-1 was further increased in the absence of HIF-1α (Supplementary Fig. S2E). This is consistent with the previously reported apparent mutual inhibition between HIF-1 and HIF-2 [11, 27].

### Basal transcription, but not transcriptional sc-heterogeneity, is altered by hypoxia

**Figure 1.**
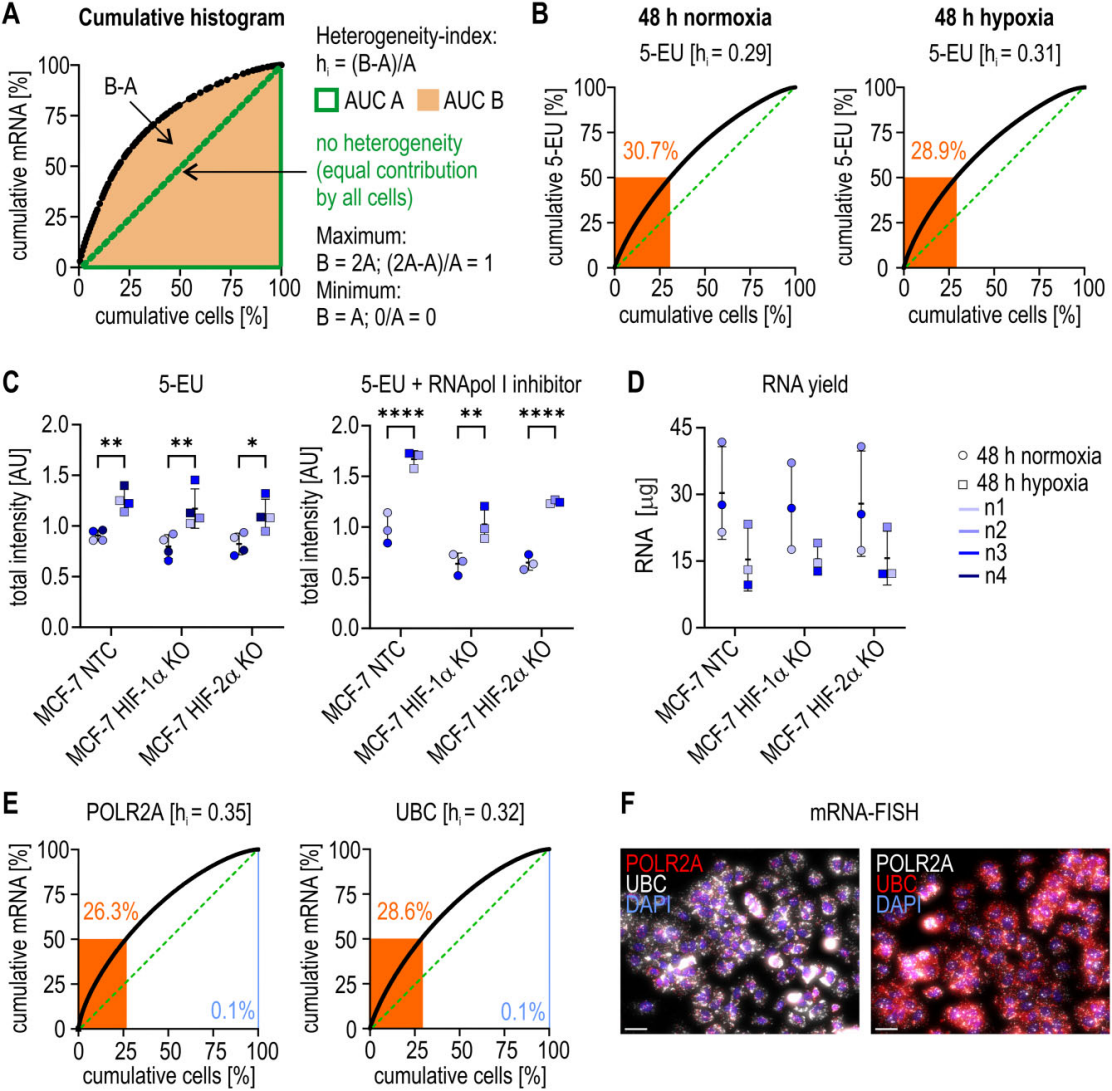
Basal transcriptional sc-heterogeneity in MCF-7 cells. (**A**) Cumulative histogram chart to calculate the heterogeneity index h_i_ as a fractional value of the AUC versus a perfectly homogenous distribution (h_i_ = 0, green line). (**B**) Representative charts of global transcriptional sc-heterogeneity as measured by 5-EU incorporation into MCF-7 NTC clone A4 cells under normoxic and hypoxic conditions. (**C**) Total 5-EU incorporation into MCF-7 NTC, HIF-1α KO, and HIF-2α KO clones A4, A3, and 2C4, respectively, in the absence or presence of low levels of actinomycin D (40 ng/ml) to specifically inhibit RNA polymerase I (RNApol I). (**D**) Yield of RNA extraction. The symbols used in panels (C) and (D) are illustrated on the right. (**E**) Representative charts of the degree of mRNA sc-heterogeneity of the housekeeping genes POLR2A and UBC in MCF-7 NTC clone A4 cells. (**F**) Fluorescence images of POLR2A and UBC mRNA-FISH in hypoxic cells, demonstrating the effects of fluorophore swapping (red, Opal 570; white, Opal 650; blue, DAPI; scale bars = 30 μm). (B, E) The percentages of cells contributing to 50% or 0% of the total mRNA are indicated.

We next defined a simple heterogeneity index h_i_ on the basis of double-cumulative histogram charts (Fig. 1A): h_i_ was calculated as a fractional value of the area under the curve (AUC), where the minimal heterogeneity (h_i_ = 0) indicates equal contribution by all cells, and the maximal heterogeneity (h_i_ = 1) indicates that a single cell alone contributes 100% of the total. To determine the basal sc-heterogeneity of global RNA transcription, 5-EU incorporation assays were performed, and images were quantified at the sc-level. A h_i_ of ∼0.3 was obtained, with ∼30% of all cells contributing to 50% of the total 5-EU (Fig. 1B). This sc-heterogeneity remained unchanged under hypoxic conditions (48 h, 0.2% O_2_). Representative fluorescence images and mean values of independent repetitive experiments are shown in Supplementary Fig. S3A. Despite a likely reduction in cellular energy metabolism, 5-EU incorporation even increased under these rather severe hypoxic conditions (Fig. 1C). The addition of low levels of actinomycin D, which specifically inhibits RNA polymerase I-mediated transcription [28], did not affect increased 5-EU incorporation, suggesting that mainly mRNA and not rRNA synthesis is increased under hypoxic conditions (Fig. 1C). In line with a previous report showing that hypoxia lowers RNA stability rather than production [29], the yield of extracted RNA from hypoxic cells was reduced (Fig. 1D). Notably, almost identical sc-variability indices were obtained after sc-quantification of the specific mRNA-FISH signals for the housekeeping genes *POLR2A* (encoding RNA polymerase II subunit A) and *UBC* (encoding ubiquitin c) (Fig. 1E; Supplementary Fig. S3B). In summary, these results define the background sc-variability of housekeeping control mRNAs, which is driven mainly by the general transcriptional sc-variability. If not indicated otherwise, all further sc-heterogeneity data will be shown only for hypoxic cells, and all further analyses will be normalized to the mean value of the cellular intensities over all cells of a POLR2A probe labeled with the same fluorophore to correct for differences in fluorescence brightness, as illustrated in Fig. 1F.

### “Inheritance” of the intrinsically high transcriptional sc-heterogeneity of the HIF pathway

**Figure 2.**
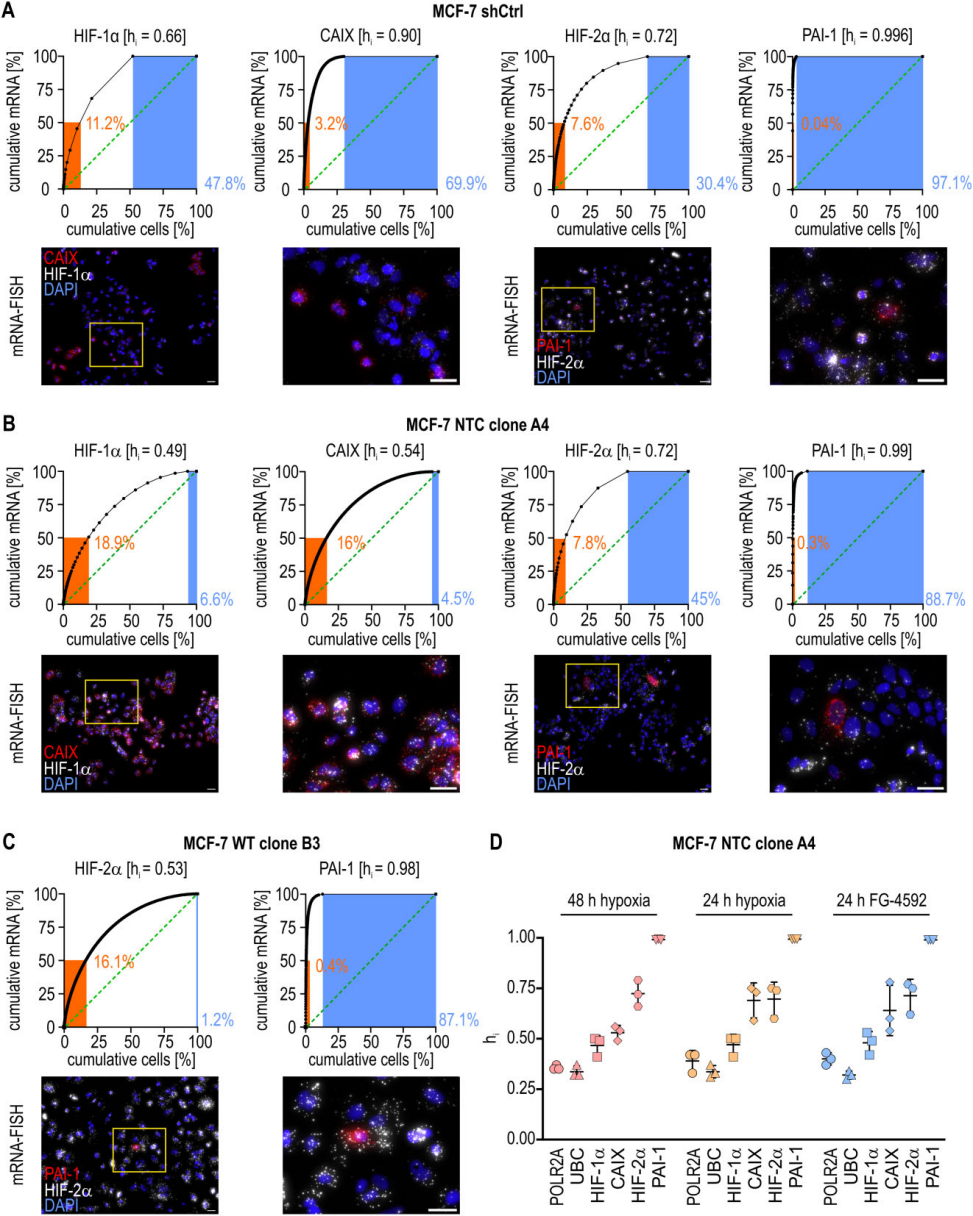
High sc-heterogeneity of HIFα isoform-specific target gene expression. Nonclonal MCF-7 shCtrl (**A**), MCF-7 NTC clone A4 (**B**), and MCF-7 WT clone B3 (**C**) cells were exposed to hypoxia for 48 h and analyzed by mRNA-FISH. The data are displayed in representative histogram charts as outlined in Fig. 1. Exemplary fluorescence microscopy images, including magnifications of the indicated regions, are displayed in the bottom panels (scale bars = 30 μm). (**D**) Overview of the sc-heterogeneity indices of the indicated mRNAs in MCF-7 NTC clone A4 cells exposed for 48 and 24 h to hypoxia or 24 h to the HIFα stabilizer FG-4592.

We first determined the sc-heterogeneity of HIF-1α and its target CAIX as well as HIF-2α and its target PAI-1 in nonclonal WT-like MCF-7 shCtrl cells (Fig. 2A). Although it is commonly accepted that HIF-1α expression is ubiquitous [30], HIF-1α mRNA sc-heterogeneity was greater than the housekeeping controls, with a higher average h_i_ of 0.62, fewer cells (∼12%) contributing to 50% of the total HIF-1α mRNA, and more cells (42%) not expressing HIF-1α at all. HIF-2α mRNA sc-heterogeneity was even somewhat higher than HIF-1α (Supplementary Fig. S4A). Unexpectedly, most hypoxic cells were negative for CAIX (∼64%) and especially PAI-1 (∼95%) mRNA with h_i_ values of 0.89 and 0.995, respectively (Supplementary Fig. S4A). Similar sc-heterogeneity data for CAIX and PAI-1 were obtained with the nonclonal MCF-7 shHIF-1α and shHIF-2α sublines, despite the knockdown of the respective HIFα isoform (Supplementary Fig. S4B).

Because nonclonal MCF-7 cells are known to display a large (epi)genetic variability, potentially explaining these findings, we next analyzed freshly cloned cells derived from two distant nonclonal MCF-7 sublines, either the commercial subline used for KO experiments or our in-house subline used for WT and knockdown experiments, resulting in the WT-like NTC clone A4 (Fig. 2B) and the WT clone B3 (Fig. 2C), respectively. While the HIF-1α/CAIX sc-heterogeneities indeed decreased (but remained higher than housekeeping genes), the PAI-1 h_i_ values in the clonal MCF-7 NTC cells were still exceedingly high with only ∼0.2% of the cells contributing 50% of the total (Fig. 2B and Supplementary Fig. 5A). These HIF-2α/PAI-1 h_i_ data were confirmed by repetitive experiments, including two additional independent MCF-7 NTC clones (Supplementary Fig. S5B) as well as two additional independent MCF-7 WT clones (Fig. 2C and Supplementary Fig. S5B).

In order to exclude potential problems with the PAI-1 mRNA-FISH probe, we additionally confirmed the rare PAI-1 expression by IF (Supplementary Fig. S5C). Therefore, to somewhat increase the PAI-1 protein levels, we used MCF-7 shHIF-1α cells exposed to hypoxia for 72 h because this was previously shown to enhance the PAI-1 mRNA and protein levels (Supplementary Fig. S2A and B).

We further considered that these large differences in HIF pathway sc-heterogeneities might be due to a loss of mRNA, inhomogeneous cell distribution, and/or regional variability in pericellular oxygen availability under prolonged hypoxic conditions [10]. However, half of the duration of hypoxia (24 h) as well as chemical stabilization of HIFα by FG-4592/roxadustat (implying a more even distribution in the culture medium than oxygen, both vertically and horizontally) basically led to the same results (Fig. 2D and Supplementary Fig. S6).

In summary, the consistent high sc-heterogeneity of the HIF-1 and especially HIF-2 pathways in nonclonal MCF-7 cells as well as in each three freshly prepared independent clonal lines derived from two distant nonclonal MCF-7 sublines, suggests “inheritance” of this hypoxic transcript pattern, even though the original single cells giving rise to these clones statistically most likely belonged to the PAI-1-negative cohort.

### HIFα mRNA expression correlates with target gene mRNA expression in breast cancer tumor samples but not in MCF-7 cells at the sc-level

**Figure 3.**
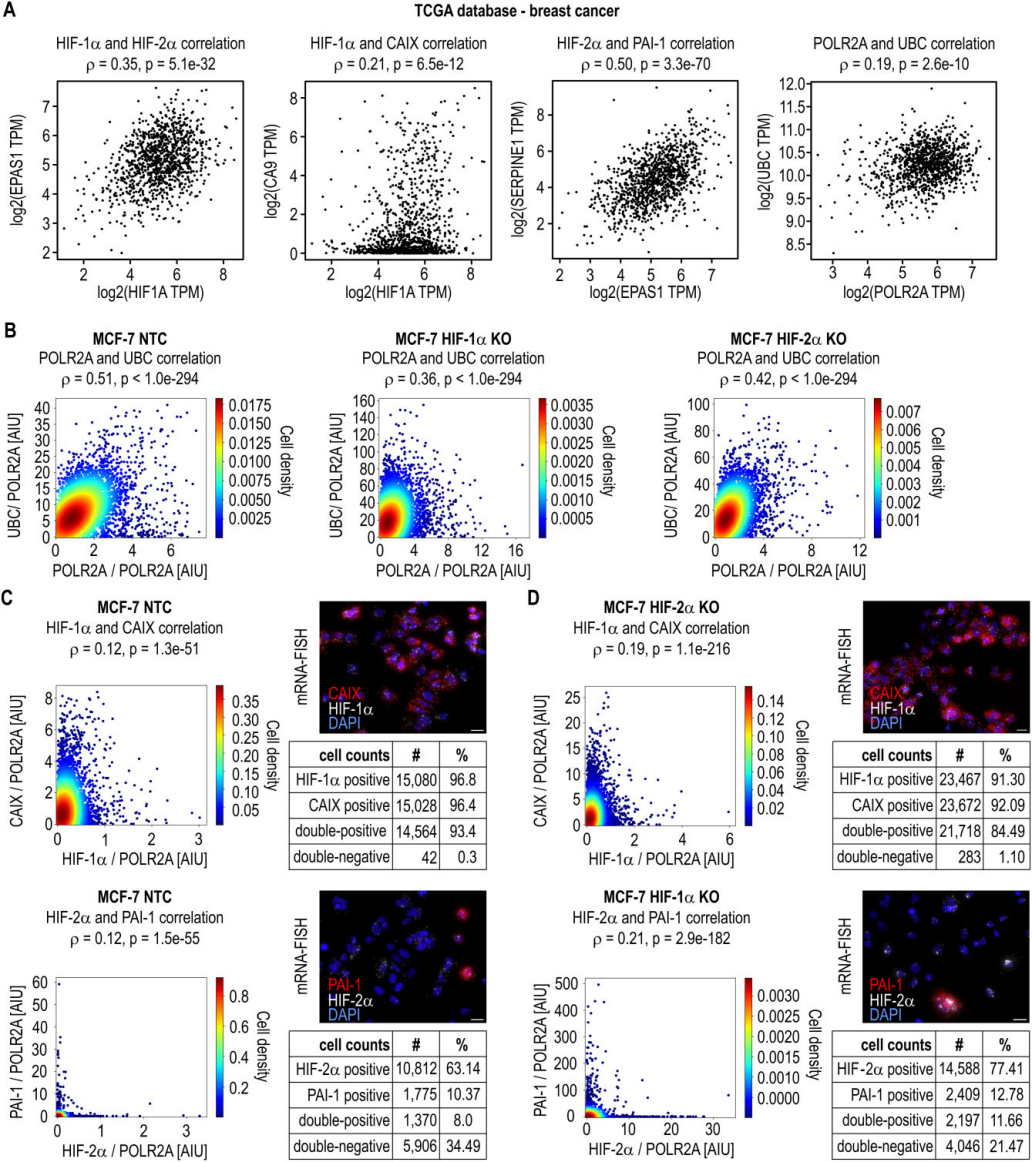
mRNA correlation analyses. (**A**) Correlation analyses of the indicated mRNAs in batch RNA-seq data of breast cancer tumor samples (TCGA database). (**B**–**D**) MCF-7 NTC clone A4 and HIF-1α KO clone A3 or HIF-2α KO clone 2C4 cells were exposed to hypoxia for 48 h, and the coexpression of the indicated genes was analyzed via mRNA-FISH at the sc-level. The fluorescence intensities of the mRNAs of interest were normalized to the average intensity per cell of POLR2A probes labeled with the same fluorophore. Data are shown in scatter plots (assembly of 3 independent experiments; *ρ*, Spearman’s correlation coefficient; AIU, arbitrary intensity unit). (B) Reference value of the correlation between the housekeeping POLR2A and UBC mRNA sc-levels. (C, D) Exemplary fluorescence microscopy images are provided in the upper panels (scale bars = 20 μm). Tables in the lower panels list the number (#) and percentage (%) of cells with the indicated mRNAs over or below the detection limit.

To clarify whether HIF target gene sc-heterogeneity is driven by the respective HIFα isoform mRNA sc-distribution, mRNA correlation analyses were employed. Correlation analyses are often performed using tumor batch RNA-seq data to identify potentially causative associations between members of the same signaling pathway, such as transcription factors and their downstream targets. Using the breast cancer mRNA data deposited in the TCGA database, we detected positive correlations between the levels of HIF-1α and HIF-2α, between HIF-1α and its target CAIX, and between HIF-2α and its target PAI-1. Notably, these HIF pathway correlations were greater than the reference values of the correlation between the housekeeping POLR2A and UBC mRNA levels (Fig. 3A).

The same pair of housekeeping genes was used to determine a control correlation for *in vitro* cultured MCF-7 NTC, HIF-1α, and HIF-2α KO cell lines. As shown in Fig. 3B, the Spearman’s correlation coefficients (*ρ*) were 0.51, 0.36, and 0.42, respectively, and served as reference values for a biologically relevant positive sc-correlation.

Among all the HIF-1α-positive (96.8% of the total) MCF-7 NTC cells, 96.6% were also CAIX mRNA positive, and the corresponding *ρ* value was only 0.12. Among all the HIF-2α-positive (63.1% of the total) cells, 12.7% were also positive for PAI-1 mRNA but again without any correlation (*ρ* = 0.12) (Fig. 3C and Supplementary Fig. S7A). In the MCF-7 HIF-2α and HIF-1α KO cell lines, the abundance of CAIX and PAI-1/HIF-2α mRNAs, respectively, was somewhat enhanced, but this did not substantially increase the correlation coefficients, which clearly remained below the housekeeping reference values (Fig. 3D and Supplementary Fig. S7A). For comparison, the estimated correlations of the nonrelated CAIX/HIF-2α, PAI-1/HIF-1α, and CAIX/PAI-1 pairs were similarly low (Supplementary Fig. S8A).

Taken together, these data suggest that mRNA correlations in tumor samples are not necessarily based on the sc-heterogeneity within the cancer cells themselves but rather reflect the average microenvironmental (hypoxic) and/or (epi)genetic conditions of each tumor sample.

### MCF-7 sc-heterogeneity analyses based on scRNA-seq

**Figure 4.**
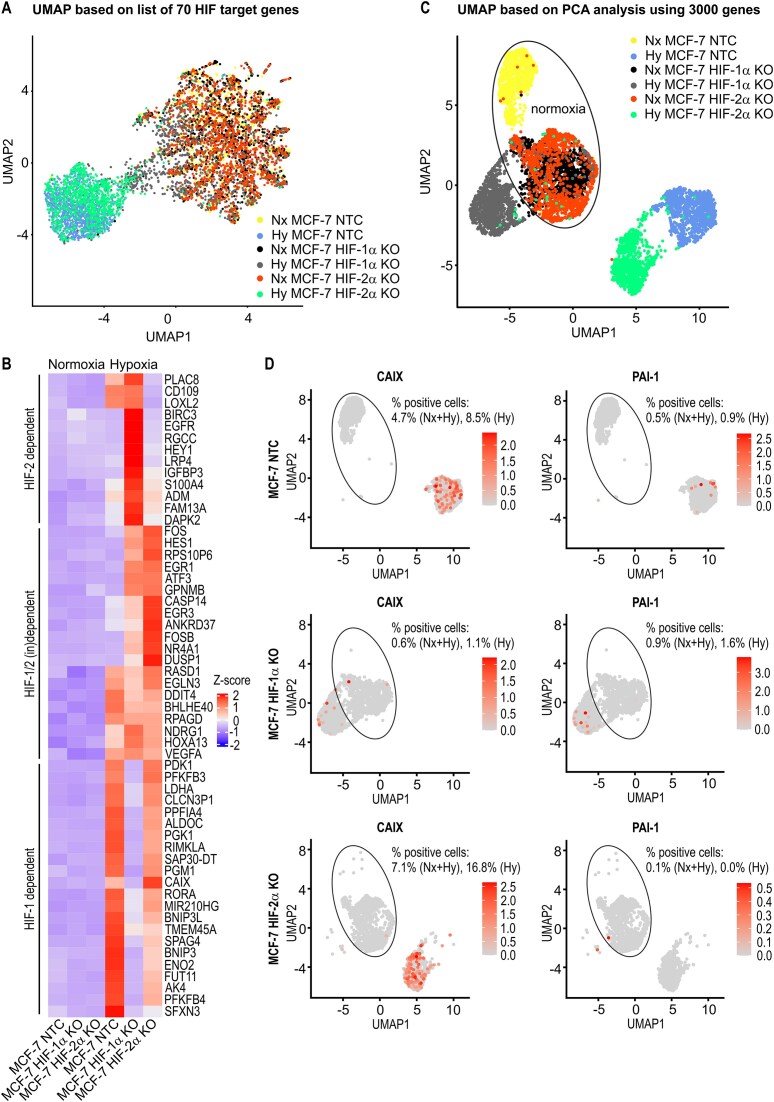
scRNA-seq of MCF-7 cells. MCF-7 NTC clone A4 and HIF-1α KO clone A3 or HIF-2α KO clone 2C4 cells were exposed to normoxia (Nx) or hypoxia (Hy) for 48 h, and subjected to scRNA-seq. (**A**) UMAP plot generated by supervised clustering of scRNA-seq data derived from 6482 cells on the basis of the list of 70 HIF isoform-specific target genes shown in Supplementary Fig. S9. Oxygenation and the different cell lines are marked by the indicated colors. (**B**). Heat map depicting the expression of the 55 top hypoxically upregulated genes with log_2_ (fold change) > 2 and minimal expression > 350 TPM. Genes are grouped according to their regulation by the specific HIFα isoforms. (**C**) UMAP plot generated by unsupervised clustering of the same scRNA-seq data on the basis of the 3000 most strongly expressed genes. (**D**) UMAP plots grouped by the indicated cell lines. The color code represents the log_2_ of the TPM. The percentages of cells positive for CAIX or PAI-1, as calculated for normoxic and hypoxic (Nx + Hy) or only hypoxic (Hy) cells, are provided. A cell containing at least one corresponding sequence read was considered positive.

To corroborate the mRNA-FISH data, we next applied scRNA-seq to MCF-7 cells cultured *in vitro*. We first performed supervised UMAP analysis using an MCF-7-derived list of 70 hypoxia-inducible and HIF-dependent genes (Supplementary Fig. S9) on combined data comprising the two conditions (normoxia and hypoxia) and the three genotypes (NTC, HIF-1α KO, and HIF-2α KO). However, only limited clustering of these six original samples was obtained, resulting in two well-separated clusters, one consisting of hypoxic cells only and the other consisting of almost all normoxic cells plus some hypoxic cells (Fig. 4A). When these clusters were further analyzed for genotype, it became evident that the “hypoxic cluster” was mainly defined by HIF-1 target genes and that the hypoxic cells overlapping with the “normoxic cluster” were mainly HIF-1α KO cells, which is consistent with the predominance of HIF-1 in the hypoxia response. While CAIX was confirmed to be a HIF-1-dependent target gene, PAI-1 mRNA was not among the top HIF target genes listed in Fig. 4B. Notably, subfractions of HIF-2- and HIF-1/2-dependent genes showed substantial hypoxic induction only in HIF-1α and HIF-1α-or-2α KO cells, respectively, but not in MCF-7 NTC cells (Fig. 4B), demonstrating that the apparent mutual inhibition between HIF-1 and HIF-2 affects only a specific set of their respective target genes.

In contrast, unsupervised UMAP analysis, which is based on PCA with the 3000 most strongly expressed genes, distinguished well between normoxic and hypoxic conditions and rederived the original six samples, except for an overlap between the normoxic HIF-1α and HIF-2α KO samples (Fig. 4C). In the cell line-specific UMAP plots, CAIX- and PAI-1-positive cells almost exclusively appeared in the hypoxic MCF-7 NTC cell cluster as well as in the HIF-2α and HIF-1α KO clusters, respectively (Fig. 4D). With 91.5% and 99.1% of all hypoxic MCF-7 NTC cells being negative for CAIX and PAI-1, respectively, the sc-heterogeneity appeared even more dramatic than that based on the results of the mRNA-FISH analyses shown in Fig. 2. When mRNA-FISH was used, only 4.6% and 89.8% of hypoxic MCF-7 NTC cells were negative for CAIX and PAI-1, respectively (Supplementary Fig. S5A), demonstrating that the low sensitivity of scRNA-seq limits such sc-heterogeneity analyses.

Overall, our data suggest that any tumor analysis solely based on a limited set of HIF target genes, as conventionally performed by immunohistochemistry of tumor biopsies, might not be sufficient to reliably identify cancer cells with a hypoxic signature. While scRNA-seq might be superior in identifying such cells, its low sensitivity leads to an inadequate reflection of the actual sc-heterogeneity.

### Widespread sc-heterogeneity of the HIF response in cancer cell lines

To consolidate our findings, we analyzed BNIP3 and EGFR, two alternative target genes of HIF-1 and HIF-2, respectively, which are relevant to cancer biology (Supplementary Fig. S8B). In hypoxic MCF-7 NTC cells, BNIP3 and EGFR also exhibited considerably high sc-heterogeneities (Supplementary Fig. S8C), clearly over the housekeeping control of h_i_ = 0.32–0.35 (Fig. 1E). Not only the correlations with their corresponding regulatory HIFα isoforms (Supplementary Fig. S8D) but even the correlations among the respective HIF-1 and HIF-2 target gene pairs BNIP3/CAIX and EGFR/PAI-1 (Supplementary Fig. S8E) remained below the housekeeping control of *ρ* ∼ 0.5 (Fig. 3B). As expected, there were no or even negative correlations between the nonrelated target gene pairs BNIP3/PAI-1, EGFR/CAIX, and BNIP3/EGFR (Supplementary Figs S7B and S8E).

**Figure 5.**
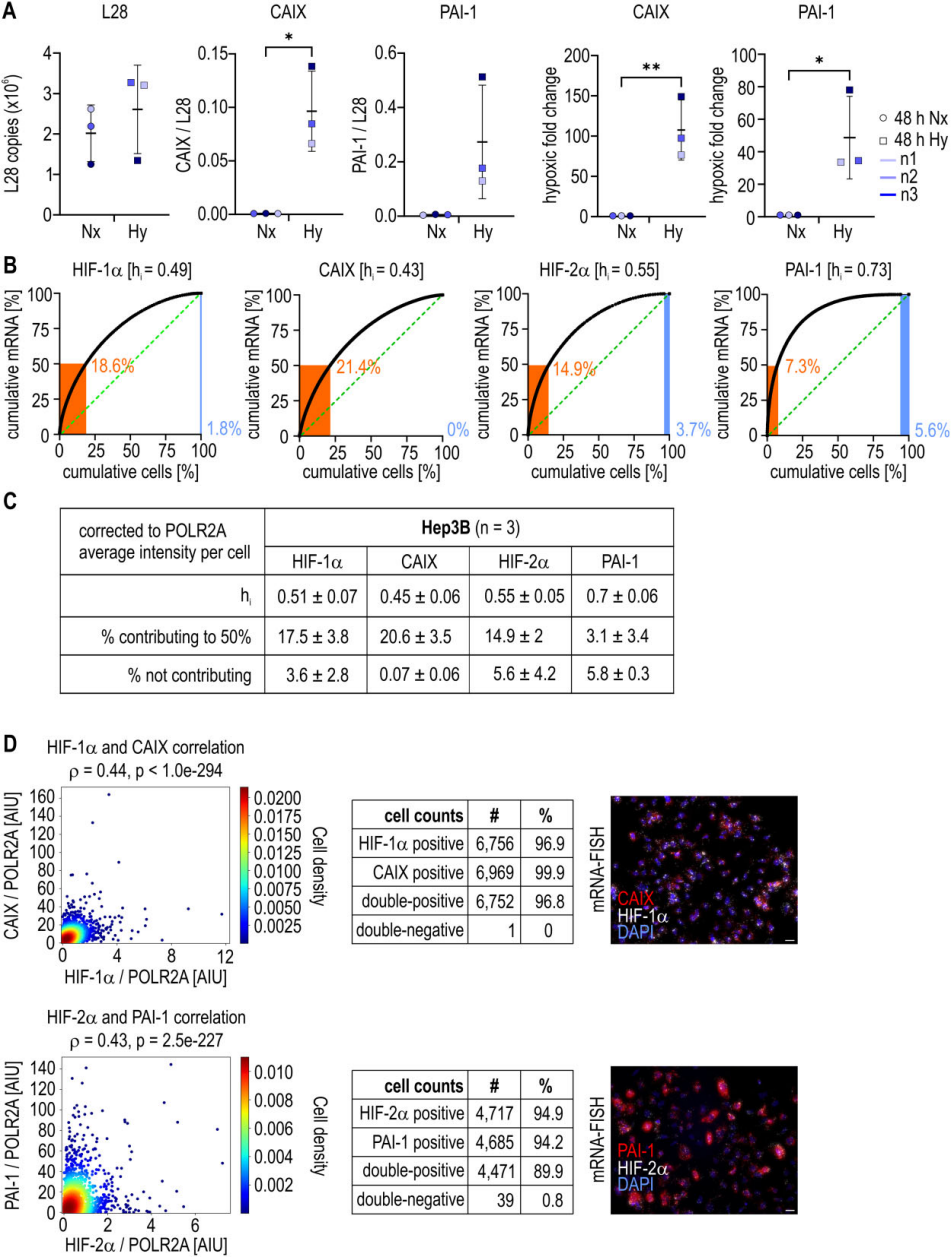
sc-Heterogeneity of the HIF response in Hep3B cells. Hep3B cells were exposed to normoxia (Nx) or hypoxia (Hy) for 48 h. (**A**) Batch transcript levels were quantified by RT-qPCR, divided by the corresponding levels of the ribosomal protein L28 mRNA, and normalized to the normoxic controls. The mean values ± SD of *n* = 3 independent experiments are shown. Unpaired *t*-test was used to statistically evaluate differences compared with the normoxic controls. *P< .05; **P< .01. (**B**, **C**) sc-Transcript levels were analyzed by mRNA-FISH and the data are displayed in representative histogram charts as outlined in Fig. 1. (B) and in a table containing assembled data (C). (**D**) Scatter plots of the sc-correlation analyses performed as in Fig. 3. Exemplary fluorescence microscopy images are shown (scale bars = 20 μm), and the number (#) and percentage (%) of cells with the indicated mRNAs over or below the detection limit are listed.

We then investigated whether the high sc-heterogeneity of the HIF pathway in MCF-7 cells is a general phenomenon. Therefore, additional cancer cell lines (Hep3B hepatoma, U2OS osteosarcoma, and MDA-MB-231 triple-negative breast adenocarcinoma) were exposed to hypoxia and analyzed as earlier. As exemplified for Hep3B cells, hepatocellular carcinoma cells also strongly induced CAIX and PAI-1 under hypoxia (Fig. 5A). While the sc-heterogeneity of HIF-1α was similar, the sc-distributions of CAIX, HIF-2α, and PAI-1 mRNAs were less heterogeneous in Hep3B cells than in MCF-7 cells (Fig. 5B). With an average of 20.6% and 3.1% of all cells contributing to 50% of the total CAIX and PAI-1 mRNAs, respectively, Hep3B cells also showed considerable sc-heterogeneity in the HIF response (Fig. 5C). Consistent with the decreased sc-heterogeneity, the HIF-1α/CAIX and HIF-2α/PAI-1 mRNA sc-correlations were greater than those in MCF-7 cells and approached the MCF-7 housekeeping control of *ρ* ∼ 0.5 (Fig. 5D).

Whereas CAIX mRNA was strongly induced by hypoxia in U2OS cells, PAI-1 induction was not significant (Supplementary Fig. S10A). Nevertheless, mRNA sc-heterogeneity was analyzed and, compared with that in MCF-7 cells, was found to be similarly high for HIF-1α and high for HIF-2α but decreased for CAIX and PAI-1 (Supplementary Fig. S10B). With an average of 29.1% and 11.1% of all cells contributing to 50% of the total CAIX and PAI-1, respectively, U2OS cells presented the least sc-heterogeneity of these mRNAs among all the cell lines analyzed (Supplementary Fig. S10C). In line with the low sc-heterogeneity of CAIX and the absence of hypoxic induction of PAI-1 (suggesting that PAI-1 is not a HIF-2 target gene in this cell line), HIF-1α/CAIX mRNA levels correlated at the sc-level, whereas HIF-2α/PAI-1 did not (Supplementary Fig. S10D).

Like MCF-7 and Hep3B cells, MDA-MB-231 cells also strongly induced CAIX and PAI-1 under hypoxia (Supplementary Fig. S11A). While the sc-distribution of HIF-1α/CAIX mRNAs was slightly more heterogeneous than that in MCF-7 cells, HIF-2α/PAI-1 sc-heterogeneity was decreased (Supplementary Fig. S11B). However, with average h_i_ indices of 0.64 and 0.66, respectively, and an average of 11.4% and 9.7% of all cells contributing to 50% of total CAIX and PAI-1 mRNAs, respectively, the sc-heterogeneity of the HIF response was also considerably high in the MDA-MB-231 cells (Supplementary Fig. S11C). Like in MCF-7 cells, the HIF-1α/CAIX and HIF-2α/PAI-1 mRNA levels did not correlate at the sc-level (Supplementary Fig. S11D).

In summary, these results support the conclusion that in cancer cells, the response of HIF-mediated target genes to hypoxia clearly shows greater sc-heterogeneity than that of housekeeping control genes, which is more pronounced for HIF-2 target genes than for HIF-1 target genes.

### sc-Correlation between nuclear HIF-1α but not HIF-2α proteins and their target mRNAs

The lack of correlation between the HIFα isoform mRNA levels and the corresponding target gene mRNA levels might at least partially be explained by a discordance between the sc-levels of HIFα mRNA and nuclear protein. Therefore, we first analyzed whether there was any hypoxic costabilization of the HIF-1α and HIF-2α proteins in the same cell. This question has not yet been systematically addressed at the sc-level because of the lack of sc-detection techniques covering the entire proteome as well as major difficulties in detecting HIF-2α. We hence established sequential IF for the detection of HIF-1α and HIF-2α. Consistent with the immunoblotting results (Supplementary Fig. S1C), we detected HIF-2α immunoreactivity not only in hypoxic but also in normoxic and HIF-2α KO cells, albeit exclusively in the extranuclear space (Supplementary Fig. S12A). While we and others previously reported on extranuclear HIF-2α, including in breast cancer tissues [26, 31–34], we cannot formally rule out that these are unspecific signals. Thus, we quantified only nuclear HIFα signals because only nuclear proteins are relevant for the transcription of target genes.

**Figure 6.**
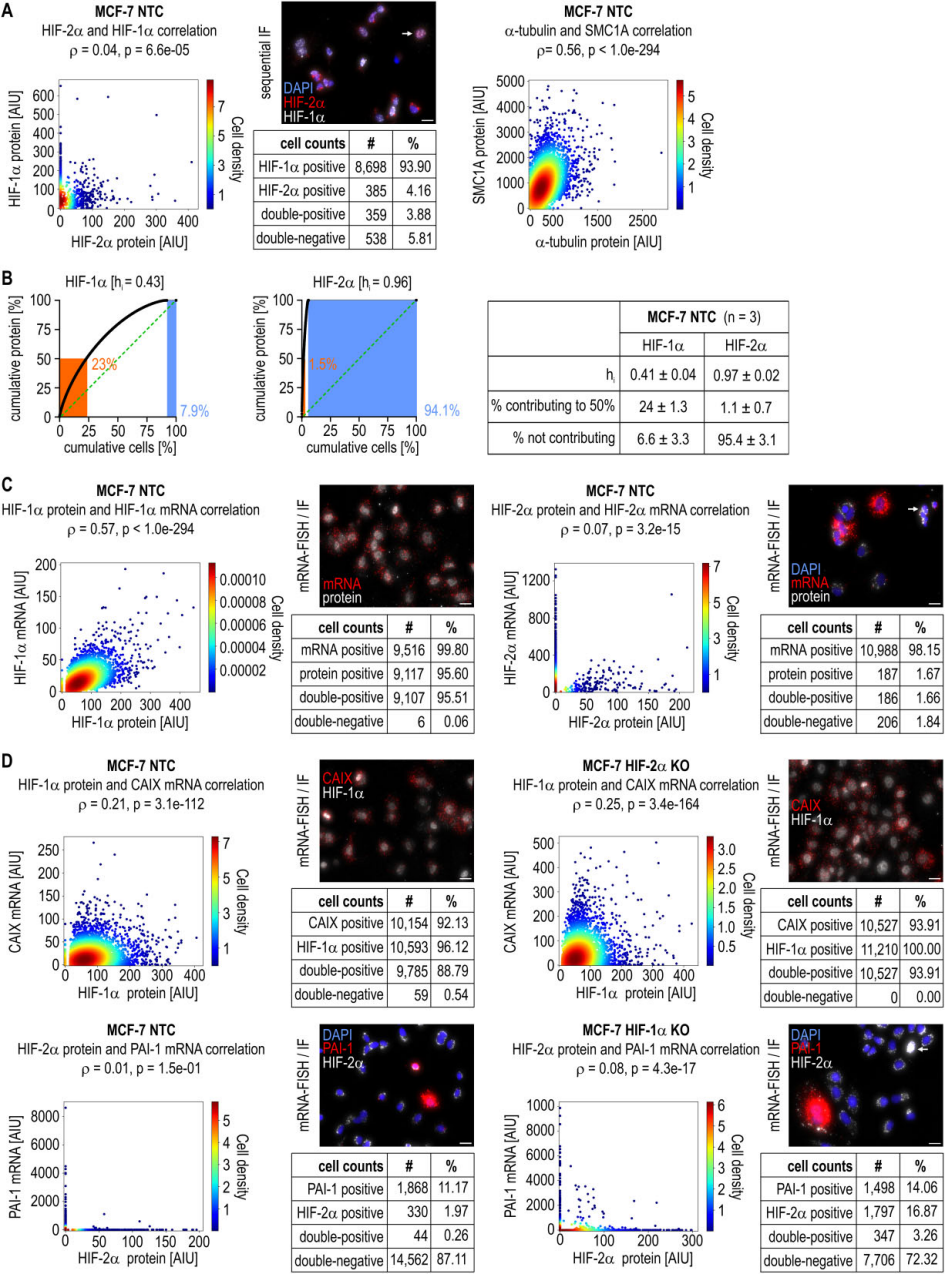
Combined mRNA and protein sc-analyses. MCF-7 NTC clone A4 and HIF-1α KO clone A3 or HIF-2α KO clone 2C4 cells were exposed to hypoxia for 48 h, fixed, and subjected to sequential IF (**A**, **B**) or mRNA-FISH followed by IF (**C**, **D**). (A, C, D) Scatter plots of the sc-correlation analyses performed as in Fig. 3, together with representative fluorescence images (scale bars = 20 μm) and tables containing assembled data. The raw fluorescence intensities of the indicated mRNAs and proteins are shown. Arrows point to cells with nuclear HIF-2α protein. (B) Representative charts and a table containing assembled data of HIF-1α and HIF-2α protein sc-heterogeneities.

While nuclear HIF-2α-positive cells were much rarer than nuclear HIF-1α-positive cells, they almost completely overlapped at the sc-level. However, there was no sc-correlation between HIF-1α and HIF-2α nuclear protein levels. For comparison, a strong sc-correlation between the two housekeeping proteins α-tubulin and structural maintenance of chromosomes protein 1A (SMC1A) was detected (Fig. 6A and Supplementary Fig. S12B). In contrast to the similar housekeeping-like sc-heterogeneities of the HIF-1α protein and mRNA, those of the HIF-2α protein-h_i_ were even greater than those of the mRNA-h_i_, with only 1.1% of all cells contributing to 50% of the total protein (Fig. 6B). As expected, the nuclear HIF-1α protein levels correlated well with its mRNA levels, but the nuclear HIF-2α protein levels did not correlate with its mRNA levels; however, the protein-positive cells fully overlapped with the corresponding mRNA-positive cells (Fig. 6C and Supplementary Fig. S12C).

Comparing the nuclear HIFα protein levels with their corresponding target gene mRNA levels, there was indeed a stronger correlation between the HIF-1α protein level and CAIX mRNA level than between the HIF-1α mRNA level and CAIX mRNA level in both MCF-7 NTC and HIF-2α KO cells. Unexpectedly, both the overlap of positive cells and the HIF-2α protein and PAI-1 mRNA correlations were even worse than the overlap and correlation between HIF-2α mRNA and PAI-1 mRNA in both MCF-7 NTC and HIF-2α KO cells (Fig. 6D and Supplementary Fig. S12D). These results were confirmed via the additional HIF-1 and HIF-2 target genes BNIP3 and EGFR, respectively. The vast majority of EGFR mRNA-positive cells were negative for nuclear HIF-2α protein, and the correlation between their levels was worse than the mRNA-mRNA correlation (Supplementary Fig. S12E).

## Discussion

While modern sc-sequencing techniques (scDNA-seq, scRNA-seq, and scATAC-seq) have provided a wealth of unprecedented information on tumor transcriptional and genetic sc-heterogeneity in recent years, the underlying mechanisms are more difficult to elucidate. “Hypoxia” is a feature that commonly arises from computer-based analyses of scRNA-seq data and is among the top differentially activated pathways in cancer tissues [35–37]. Considering the many causes of imbalanced tumor oxygenation (abnormal tumor vascularization and perfusion) and oxygen consumption (inflammation, dysregulated proliferation, and metabolic hyper- or hypoactivity), it seems obvious that tumor microenvironmental heterogeneity is responsible for the differential activation of the hypoxia pathway. However, we first noticed sc-heterogeneity in the hypoxic expression of the gene encoding erythropoietin (*Epo*; a HIF-2-specific target gene) when we observed an unexpected partial lack of Epo in otherwise Epo-capable cells in the kidneys of Epo-reporter mice [38–40]. This pattern resembled stochastic “on-off” transcription and probably contributed to the loss of Epo expression in immortalized cell lines derived from these mice [38, 41], demonstrating that it is not only the tissue pO_2_ that affects Epo expression. Herein, we have shown that there is also a considerable degree of intrinsic transcriptional sc-heterogeneity in the HIF pathway in commonly used cancer cell lines, suggesting that strong sc-heterogeneity is a common feature of the HIF response.

We reasoned that the expression of especially HIF-2-dependent genes, which are hypoxically induced in a small subfraction of all nonclonal cells, would be completely lost following cloning because the probability of deriving clones from exactly those few positive cells is negligible. Surprisingly, all clones that we analyzed maintained a similar (small) sc-frequency of HIF-2α mRNA and target gene induction, suggesting “inheritance” of intrinsic sc-heterogeneity. One potential explanation for this unexpected result might be the well-known but poorly characterized mutual inhibition between HIF-1α and HIF-2α [11, 27]. However, while HIF-2α mRNA and its target genes were indeed increased in HIF-1α knockdown and KO cells, they still showed strong sc-heterogeneity. Another explanation might be that some HIF target genes display high transcriptional burst frequencies, which are known to cause sc-variability [42]. These bursts are encoded mainly by enhancers [43, 44], and the hypoxia response element might hence affect burst frequency [45]. Unfortunately, with the techniques applied in our work, longitudinal sc-quantification of gene expression in the same cell was not possible. Therefore, we theoretically may have missed the proper time point of expression of specific genes in the majority of single cells.

Technically, we focused on mRNA-FISH rather than scRNA-seq because of the higher sensitivity and throughput when analyzing single gene products in a large number of cells. Furthermore, our scRNA-seq data demonstrated that supervised UMAP approaches based on known HIF target genes fail to cluster cells derived from well (normoxic) and poorly (hypoxic) oxygenated environments. Owing to the supposedly low complexity of a clonal cell line with only two environmental states (normoxia and hypoxia) and three genetic states (WT, HIF-1α, and HIF-2α KO), we initially assumed that this UMAP approach would recapitulate the global relationship between these cell states. However, consistent with the known failure of UMAP and other dimension reduction algorithms in recapitulating global states [46], proper clustering was obtained only following PCA using 3000 expressed transcripts. These results support the notion that the sc-heterogeneity of HIF target gene expression prevents the unambiguous identification of hypoxic cancer cells in tumor samples.

Considering that batch mRNA levels of HIFα and its target genes typically correlate in tumor samples, the low to nonexistent correlation between the sc-levels of the mRNAs of the HIFα isoforms and the corresponding target genes was an unexpected finding, indicating that in cancerous tissues scRNA-seq-based correlation analyses between HIFs and genes of interest are not necessarily suitable for identifying novel HIF target genes. A likely explanation might be that the nuclear HIFα protein levels (responsible for transactivation activity) are not in phase with the HIFα mRNA levels. In support of this hypothesis, we and others have previously shown that HIF-mediated PHD2 and PHD3 transactivation in turn decreases HIFα protein stability and hence HIF transcriptional activity even under ongoing hypoxic conditions [47–50]. Furthermore, HIF-1α (HIF-2α was not analyzed) shows a lactate-driven oscillatory expression pattern in hypoxic cancer cells, which predicts low survival of breast cancer patients [51, 52]. HIF-1α was also found to transiently increase during the G1 phase of the cell cycle [53]. In the present study, we found a clear correlation between HIF-1α mRNA and nuclear protein levels, but there was no such correlation between HIF-2α mRNA and nuclear protein levels. While this discordance could explain the lack of HIF-2α/target mRNA–mRNA correlation, the actual HIF-2α/target protein-mRNA correlation was even worse. In conclusion, we have no explanation for the unexpectedly strong sc-heterogeneity of the HIF pathway. We may only speculate on the sc-heterogeneity of additional (unknown) regulatory factors involved in HIF target gene expression, but this would still not explain the underlying mechanism. Another possibility may be very high turnover rates of HIFα protein stability and/or on/off kinetics of target gene DNA binding [54], which cannot be resolved by single time-point analyses such as those used in our study.

Because of their aberrant expression in hypoxic solid tumors, HIFs and their downstream genes are interesting targets for tumor therapy [55, 56]. Currently, there is only one drug that directly targets the HIFα subunit in clinical use: the HIF-2 dimerization inhibitor belzutifan, which is used to treat HIF-2 prevalent VHL-deficient clear cell renal cell carcinoma [57]. While this specific cancer type might be a notable exception, on the basis of our data, most cancer types appear to possess intrinsic HIFα isoform sc-heterogeneity, implying that either an unspecific HIF heterodimerization inhibitor or a combination therapy targeting both HIF-1 and HIF-2 will be necessary to successfully treat hypoxic tumor cells. Owing to their remarkable sc-heterogeneities, targeting HIF downstream genes may be of limited efficiency, even in HIFα-positive cancer cells.

## Supplementary Material

zcaf021_Supplemental_Files

## Data Availability

All materials and underlying raw data can be obtained from the corresponding author on reasonable request. The scRNA-seq data have been deposited in NCBI’s Gene Expression Omnibus and are accessible through GEO Series accession number GSE292771 (release date 29 March 2025; https://www.ncbi.nlm.nih.gov/geo/query/acc.cgi?acc=GSE292771). All primary data have been deposited in Dryad and are available via https://doi.org/10.5061/dryad.z08kprrr3 (Figs 1–6) and https://doi.org/10.5061/dryad.5x69p8dg4 (Supplementary Figs S1–S12).
