# Supplementary material for: Inherent single-cell heterogeneity of the transcriptional response to hypoxia in cancer cells
Source: NAR Cancer. 2025 Aug 28;7(3):zcaf021. doi: 10.1093/narcan/zcaf021 (PMC12409416; doi:10.1093/narcan/zcaf021)
Supplement: zcaf021_Supplemental_Files [file zcaf021_supplemental_files.zip › NARC-2025-022_revised_Supplement.pdf]

Inherent single-cell heterogeneity of the transcriptional response to hypoxia in cancer cells

Małgorzata Wilk, Thomas Knöpfel, Stana M. Burger, Stellor Nlandu Khodo, Roland H. Wenger

Institute of Physiology, University of Zürich, CH-8057 Zürich, Switzerland

SUPPLEMENTARY MATERIAL

Supplementary Table S1. Oligonucleotide sequences.

Primers for CRISPR-Cas9 target locus amplification	
HIF1A-Fwd	5’-tgtgagagttccttatgtgtgca-3’
HIF1A-Rev	5’-tcaaaacattgcgaccacct-3’
EPAS1-Fwd	5’-cccatgtgaagccctgttct-3’
EPAS1-Rev	5’-acctgaggagaggagcttgt-3’

Primers for RT-qPCR mRNA quantification	
Hs-HIF1α-Fwd	5'-tccgatggaagcactagaca-3'
Hs-HIF1α-Rev	5'-tggtgacaactgatcgaa-3'
Hs-EPAS1-Fwd	5'-ttgatgtggaaacggatgaa-3'
Hs-EPAS1-Rev	5'-ggaacctgctcttgcctgttc-3'
Hs-CAIX-Fwd	5'-gggtgtcatctggactgtgtt-3'
Hs-CAIX-Rev	5'-cttctgtgctgccttctcatc-3'
Hs-BNIP3-Fwd	5'-aactgcacttcagcaataatgg-3'
Hs-BNIP3-Rev	5'-ccgacttgaccaatcccata-3'
Hs-PAI1-Fwd	5'-actggaaaggcaacatgacc-3'
Hs-PAI1-Rev	5'-gaggaagggtctgtccatga-3'
Hs-EGFR-Fwd	5'-cagcgctaccttgtcattca-3'
Hs-EGFR-Rev	5'-ctgagctgtatcgctgcaag-3'
Hs-L28-Fwd	5'-gcaattccttccgctacaac-3'
Hs-L28-Rev	5'-tgttcttgcggatcatgtgt-3'

Supplementary Table S2. mRNA-FISH probes.

RNAscope probe	Accession No.	Target region	Channel
Hs-HIF1A	NM_001243084.1	454 - 1443	C1/C3
Hs-EPAS1	NM_001430.4	1332 - 2354	C1/C3
Hs-CA9	NM_001216.2	326 - 1528	C1
Hs-BNIP3	NM_004052.4	25 - 1398	C1
Hs-SERPINE1	NM_001165413.2	764 - 1752	C1
Hs-EGFR	NM_005228.3	505 - 1737	C1
Hs-POLR2A	NM_000937.4	2514 - 3433	C1
Hs-UBC	NM_021009	342 - 1503	C3
bacterial dapB	not provided	not provided	C1/C2/C3

Supplementary Table S3. Antibodies used for immunofluorescence.

Antigen	Type	Number	Dilution	Company
HIF-1α	rabbit monoclonal	ab179483	1:200	Abcam, Cambridge, UK
HIF-2α	rabbit monoclonal	BL-95-1A2	1:200	Bethyl Laboratories, Montgomery, TX, USA
PAI-1	mouse monoclonal	MA5-17171	1:500	Thermo Fisher Scientific, Waltham, USA
α-Tubulin	rabbit polyclonal	2144	1:200	Cell Signaling, Danvers, MA, USA
SMC1A	rabbit polyclonal	ab9262	1:200	Abcam, Cambridge, UK
Secondary goat anti-rabbit coupled to Alexa Fluor 647		A-21244	1:1,250	Thermo Fisher Scientific, Waltham, USA

Supplementary Table S4. Antibodies used for immunoblotting.

Antigen	Type	Number	Dilution	Company
HIF-1α	mouse monoclonal	Clone 54	1:750	BD Biosciences, Allschwil, Switzerland
HIF-2α	rabbit polyclonal	PAB12124	1:1,000	Abnova, Taipeh, Taiwan
CAIX	mouse monoclonal	Clone M75	1:500	Absolute Antibody, Oxford, UK
PAI-1	mouse monoclonal	MA5-17171	1:500	Thermo Fisher Scientific, Waltham, USA
β-actin	mouse monoclonal	A5441	1:10,000	Sigma Aldrich, Burlington, MA, USA
Secondary goat anti-mouse		31430	1:2,500	Thermo Fisher Scientific, Waltham, USA
Secondary goat anti-rabbit coupled to horseradish peroxidase		31460	1:2,500	Thermo Fisher Scientific, Waltham, USA

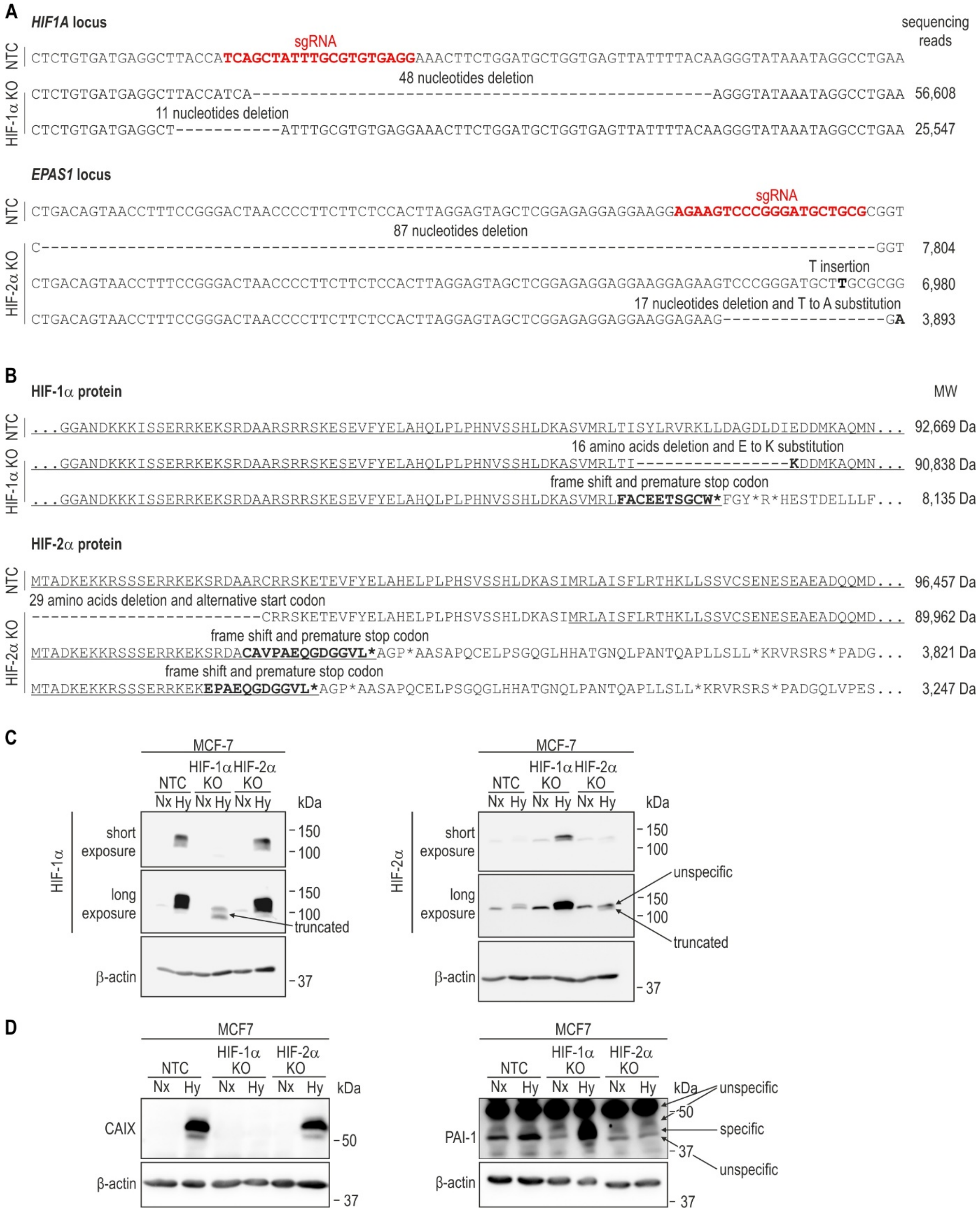

Supplementary Fig. S1. Biallelic knockout of HIF-1α or HIF-2α in MCF-7 cells. (**A**) MCF-7 cells were gene edited via CRISPR-Cas9 and single guide RNA (sgRNA) with either nontargeting control (NTC) or targeting *HIF1A* (HIF-1α KO) or *EPAS1* (HIF-2α KO) sequences as indicated (red). Following cloning, genomic DNA was extracted from the HIF-1α KO and HIF-2α KO clones A3 and 2C4, respectively, and analyzed by deep sequencing. The number of sequence reads for each KO allele is indicated. (**B**) Predicted HIF-1α and HIF-2α protein alterations in the KO clones. The translation products (underlined), deletions (dashed), substitutions (bold) and stop codons (asterisks) are indicated. The predicted molecular weights (MWs) of the translation products are provided on the right. (**C**) Immunoblotting of HIF-1α and HIF-2α using cell extracts derived from MCF-7 NTC clone A4 and

HIF-1α KO and HIF-2α KO clones A3 and 2C4, respectively, after exposure for 48 hours to normoxia (Nx) or 0.2% O_2 hypoxia (Hy). β-Actin served as a loading and blotting control. (**D**) Immunoblotting of CAIX and PAI-1 as outlined above.

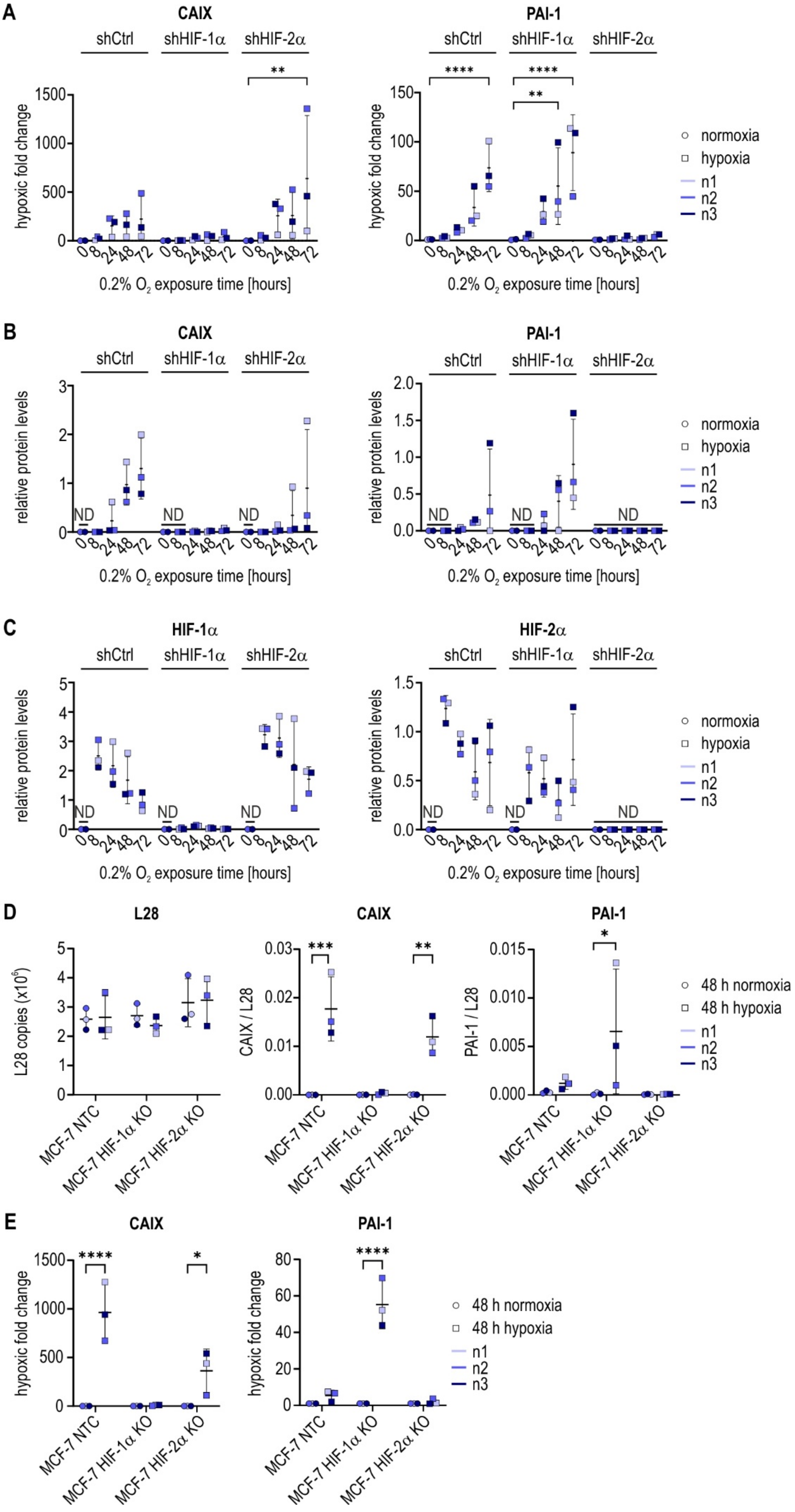
A
CAIX
shCtrl
shHIF-1α
shHIF-2α
hypoxic fold change
0.2% O2 exposure time [hours]
PAI-1
normoxia
hypoxia
n1
n2
n3
B
relative protein levels
ND
C
HIF-1α
HIF-2α
D
L28
L28 copies (x10^6)
CAIX / L28
PAI-1 / L28
MCF-7 NTC
MCF-7 HIF-1α KO
MCF-7 HIF-2α KO
48 h normoxia
48 h hypoxia
E

Supplementary Fig. S2. HIFα isoform-specific target gene induction. (**A**) Kinetics of hypoxic CAIX and PAI-1 mRNA induction in nonclonal MCF-7 shCtrl, shHIF-1α, and shHIF-2α cell cultures. Batch transcript levels were quantified via RT-qPCR, divided by the corresponding levels of the ribosomal protein L28 mRNA, and normalized to the normoxic controls. (**B**) Kinetics of CAIX and PAI-1 protein levels in nonclonal MCF-7 shCtrl, shHIF-1α, and shHIF-2α cell cultures. Protein levels were determined by immunoblotting and divided by the corresponding levels of β-actin protein. (**C**) Kinetics of HIF-1α and HIF-2α protein levels determined as in **B**. (**D**, **E**) MCF-7 NTC clone A4 and biallelic HIF-1α KO and HIF-2α KO clones A3 and 2C4, respectively, were exposed to normoxic or hypoxic conditions for 48 hours. Transcript levels were quantified via RT-qPCR, divided by the indicated ribosomal protein L28 mRNA levels (**D**), and normalized to the corresponding normoxic controls (**E**). The mean values ± S.D. of n = 3 independent experiments are shown (ND, not detectable). Two-way ANOVA followed by Bonferroni's multiple comparisons test was used to statistically evaluate differences compared with the normoxic controls. *p<0.05; **p<0.01; ***p<0.001; ****p<0.0001.

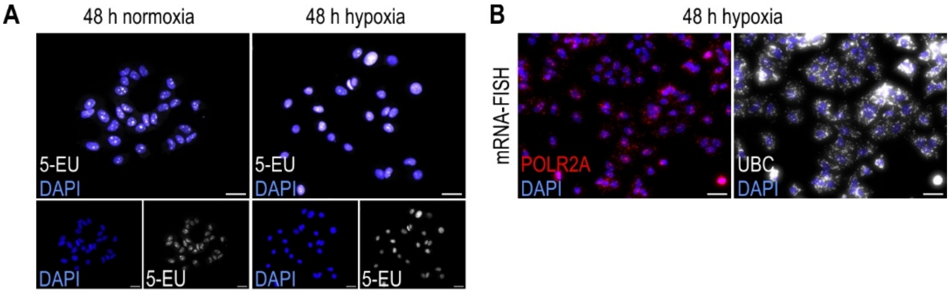

raw total intensity values	**5-EU** (n = 4)	
	48 h Nx	48 h Hy
h_i	0.29 ± 0.03	0.32 ± 0.02
% contributing to 50%	30.7 ± 1.9	28.3 ± 1.4
% not contributing	0.0 ± 0.0	0.0 ± 0.0

corrected to POLR2A average intensity per cell	**MCF-7 NTC** (n = 3)	
	POLR2A	UBC
h_i	0.36 ± 0.01	0.34 ± 0.03
% contributing to 50%	25.9 ± 0.8	27.9 ± 2.5
% not contributing	0.2 ± 0.2	0.4 ± 0.5

Supplementary Fig. S3. 5-EU incorporation and housekeeping mRNA sc-heterogeneities. (**A**) MCF-7 NTC (clone A4) cells were exposed to normoxic (Nx) or hypoxic (Hy) conditions for 48 hours, and the sc-heterogeneity (h_i) of 5-EU incorporation was determined by quantitative fluorescence microscopy (white, 5-EU; blue, DAPI; scale bars = 30 μm). (**B**) POLR2A (red) and UBC (white) mRNA sc-heterogeneities of hypoxic MCF-7 NTC (clone A4) cells (scale bars = 30 μm). sc-Heterogeneities (h_i) are listed as the means ± S.D. (n, number of independent repetitions). The percentages of cells contributing 50% (orange) or 0% (blue) of the total are indicated.

A

corrected to POLR2A (CAIX, PAI-1) or UBC (HIF-1α, HIF-2α) average intensity	**MCF-7 shCtrl** (n = 3)			
	HIF-1α	CAIX	HIF-2α	PAI-1
h_i	0.62 ± 0.1	0.89 ± 0.01	0.68 ± 0.06	0.995 ± 0.002
% contributing to 50%	12 ± 3.1	3.4 ± 0.4	9.1 ± 2.2	0.1 ± 0.9
% not contributing	42 ± 17	63.6 ± 5.6	20.2 ± 13.9	95.1 ± 1.7

B

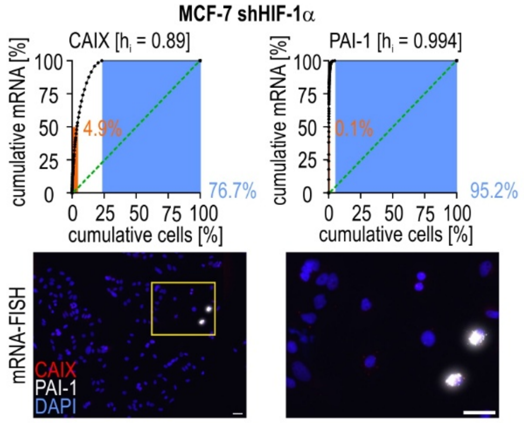

corrected to POLR2A (CAIX) or UBC (PAI-1) average intensity	**MCF-7 shHIF-1α** (n = 3)	
	CAIX	PAI-1
h_i	0.90 ± 0.07	0.994 ± 0.001
% contributing to 50%	3.7 ± 2.2	0.1 ± 0
% not contributing	77.2 ± 18.1	95.6 ± 1.2

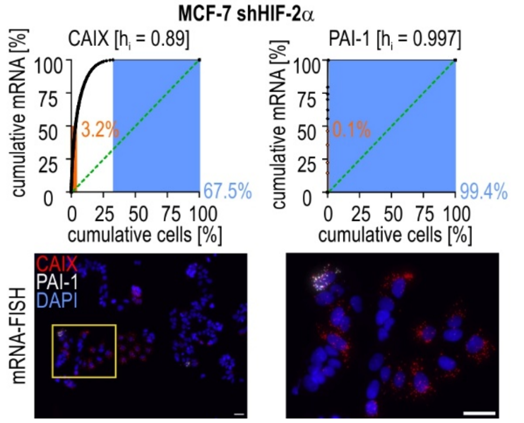

corrected to POLR2A (CAIX) or UBC (PAI-1) average intensity	**MCF-7 shHIF-2α** (n = 3)	
	CAIX	PAI-1
h_i	0.89 ± 0.01	0.996 ± 0.003
% contributing to 50%	3.3 ± 0.4	0.1 ± 0.08
% not contributing	67.9 ± 1.2	96.9 ± 4.8

Supplementary Fig. S4. mRNA sc-heterogeneities of nonclonal MCF-7 shRNA knockdown cells. MCF-7 shCtrl, shHIF-1α, and shHIF-2α cells were exposed to hypoxic conditions for 48 hours. HIF-1α, CAIX, HIF-2α and PAI-1 mRNA sc-heterogeneities were determined by quantitative fluorescence microscopy. (**A**) MCF-7 shCtrl sc-heterogeneity indices (h_i) are listed as the means ± S.D. (n, number of independent repetitions). (**B**) CAIX and PAI-1 mRNA h_i values of hypoxic MCF-7 shHIF-1α and shHIF-2α cells. The percentages of cells contributing 50% (orange) or 0% (blue) of the total are indicated. An exemplary mRNA-FISH image of CAIX (red) and PAI-1 (white) is shown (nuclei, blue; scale bars = 30 μm).

A

corrected to POLR2A average intensity	MCF-7 NTC clone A4 (n = 3)			
	HIF-1α	CAIX	HIF-2α	PAI-1
h_i	0.47 ± 0.05	0.53 ± 0.04	0.72 ± 0.07	0.991 ± 0.002
% contributing to 50%	20.1 ± 2.7	16.9 ± 1.7	8 ± 1.4	0.2 ± 0.1
% not contributing	4.5 ± 2.8	4.6 ± 3.5	39.3 ± 21.4	89.8 ± 5

B

corrected to POLR2A average intensity	MCF-7 NTC clones (n = 1)					
	HIF-2α			PAI-1		
	A4	C2	B2	A4	C2	B2
h_i	0.70	0.82	0.65	0.997	0.97	0.98
% contributing to 50%	7.7	4.8	9.7	0.02	0.4	0.3
% not contributing	21.4	57.0	10.2	96.9	88.8	90.6

corrected to POLR2A average intensity	MCF-7 WT clones (n = 1)					
	HIF-2α			PAI-1		
	A8	B3	C4	A8	B3	C4
h_i	0.55	0.53	0.55	0.98	0.98	0.98
% contributing to 50%	14.9	16.1	14.7	0.4	0.4	0.7
% not contributing	0.8	3.4	0.7	87.8	87.1	80.9

corrected to POLR2A average intensity	MCF-7 NTC clones (n = 3 independent clones)		MCF-7 WT clones (n = 3 independent clones)	
	HIF-2α	PAI-1	HIF-2α	PAI-1
h_i	0.72 ± 0.09	0.98 ± 0.01	0.54 ± 0.01	0.98 ± 0.0
% contributing to 50%	7.4 ± 2.5	0.2 ± 0.2	15.2 ± 0.8	0.5 ± 0.2
% not contributing	29.5 ± 24.4	92.1 ± 4.3	0.9 ± 0.3	85.3 ± 3.8

C

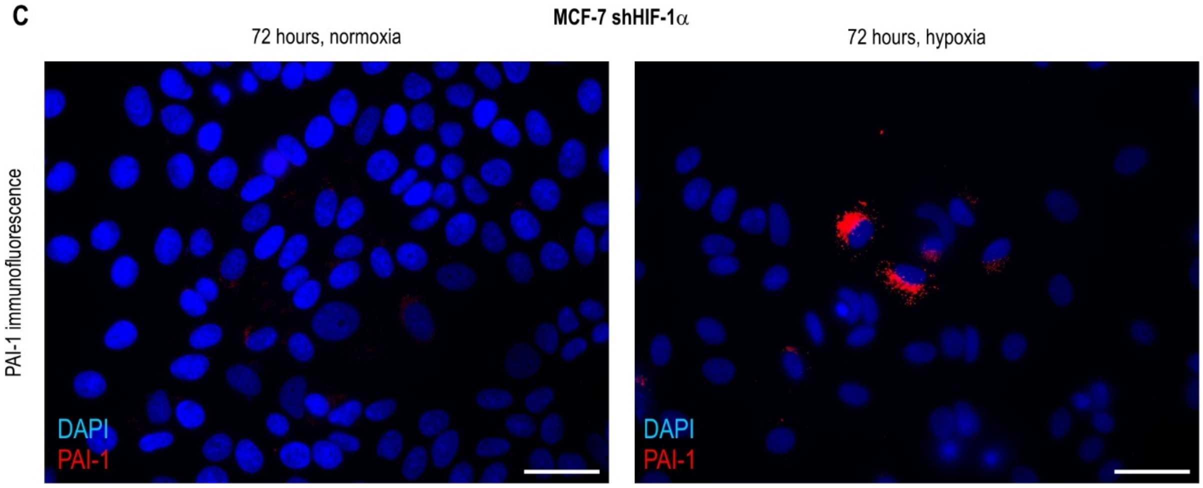

Supplementary Fig. S5. mRNA sc-heterogeneities of clonal MCF-7 NTC and WT cells.
(**A**) HIF-1α, CAIX, HIF-2α and PAI-1 mRNA sc-heterogeneities of hypoxic MCF-7 NTC clone A4 cells. (**B**) mRNA sc-heterogeneities mRNA sc-heterogeneities (h_i) in each three independent clones derived from the company's (NTC) or our in-house (WT) MCF-7 sublines were analyzed by quantitative fluorescence microscopy, and are listed as the means ± S.D. (n, number of independent repetitions). (**C**) PAI-1 immunfluorescence (red) of nonclonal MCF-7 shHIF-1α cells exposed to normoxia or hypoxia (0.2% O_2) for 72 hours (nuclei, blue; scale bars = 30 μm).

A

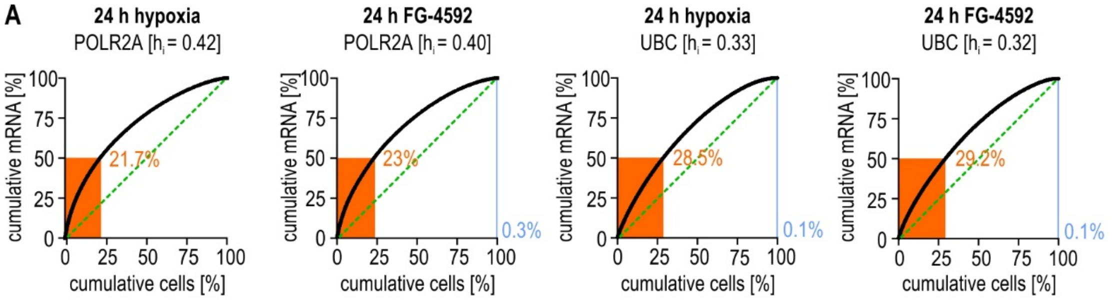

corrected to POLR2A average intensity	MCF-7 NTC clone A4 (n = 3)			
	POLR2A		UBC	
	24 h Hy	24 h FG-4592	24 h Hy	24 h FG-4592
h_i	0.39 ± 0.05	0.4 ± 0.03	0.34 ± 0.03	0.32 ± 0.02
% contributing to 50%	23.5 ± 3.5	22.5 ± 2.2	28.2 ± 1.5	29 ± 0.5
% not contributing	0.03 ± 0.06	0.2 ± 0.1	0.03 ± 0.06	0.1 ± 0.1

B

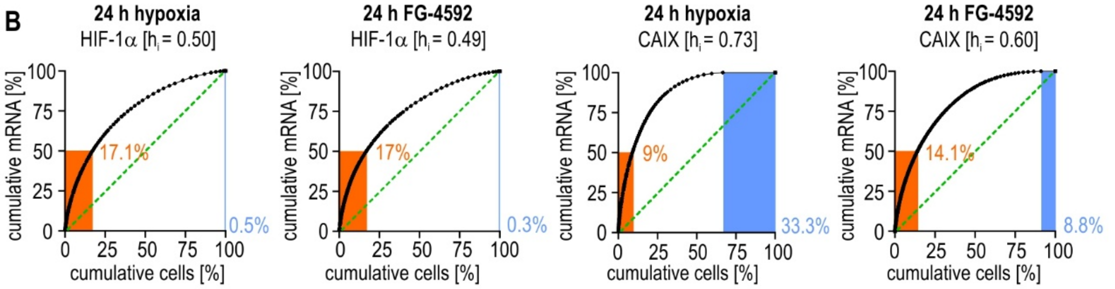

corrected to POLR2A average intensity	MCF-7 NTC clone A4 (n = 3)			
	HIF-1α		CAIX	
	24 h Hy	24 h FG-4592	24 h Hy	24 h FG-4592
h_i	0.47 ± 0.05	0.48 ± 0.06	0.69 ± 0.09	0.64 ± 0.1
% contributing to 50%	18.7 ± 3	18.1 ± 3.5	10.5 ± 3.3	12.1 ± 4.8
% not contributing	0.6 ± 0.5	0.4 ± 0.5	26.6 ± 15.6	25.2 ± 17.5

C

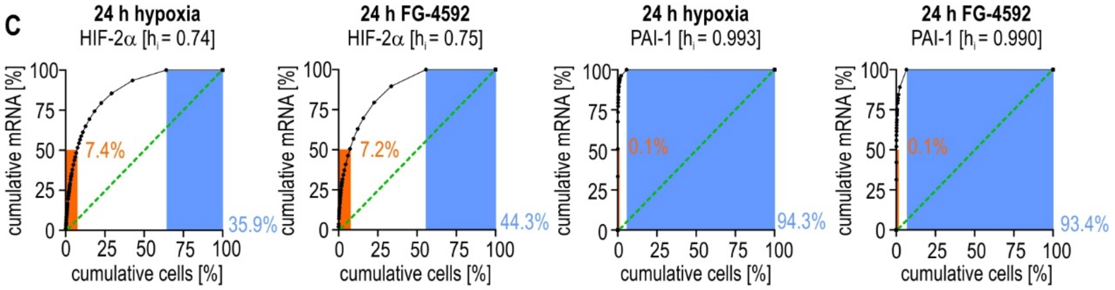

corrected to POLR2A average intensity	MCF-7 NTC clone A4 (n = 3)			
	HIF-2α		PAI-1	
	24 h Hy	24 h FG-4592	24 h Hy	24 h FG-4592
h_i	0.70 ± 0.08	0.71 ± 0.08	0.994 ± 0.001	0.990 ± 0.0
% contributing to 50%	8.5 ± 3.3	8.7 ± 3.5	0.1 ± 0.0	0.2 ± 0.06
% not contributing	26.1 ± 10.5	34.5 ± 10.5	94 ± 1.3	92.6 ± 1.9

Supplementary Fig. S6. mRNA sc-heterogeneities following short-term hypoxia and chemical HIFα stabilization. MCF-7 NTC clone A4 cells were exposed to hypoxic (Hy) conditions or to FG-4592/roxadustat for 24 hours, and the sc-heterogeneities (h_i) of the indicated housekeeping (**A**), HIF-1 (**B**) or HIF-2 (**C**) pathway mRNAs were determined. sc-Heterogeneity indices are shown as representative histogram charts and are listed as the mean ± S.D. (n, number of independent repetitions). The percentages of cells contributing 50% (orange) or 0% (blue) of the total are indicated.

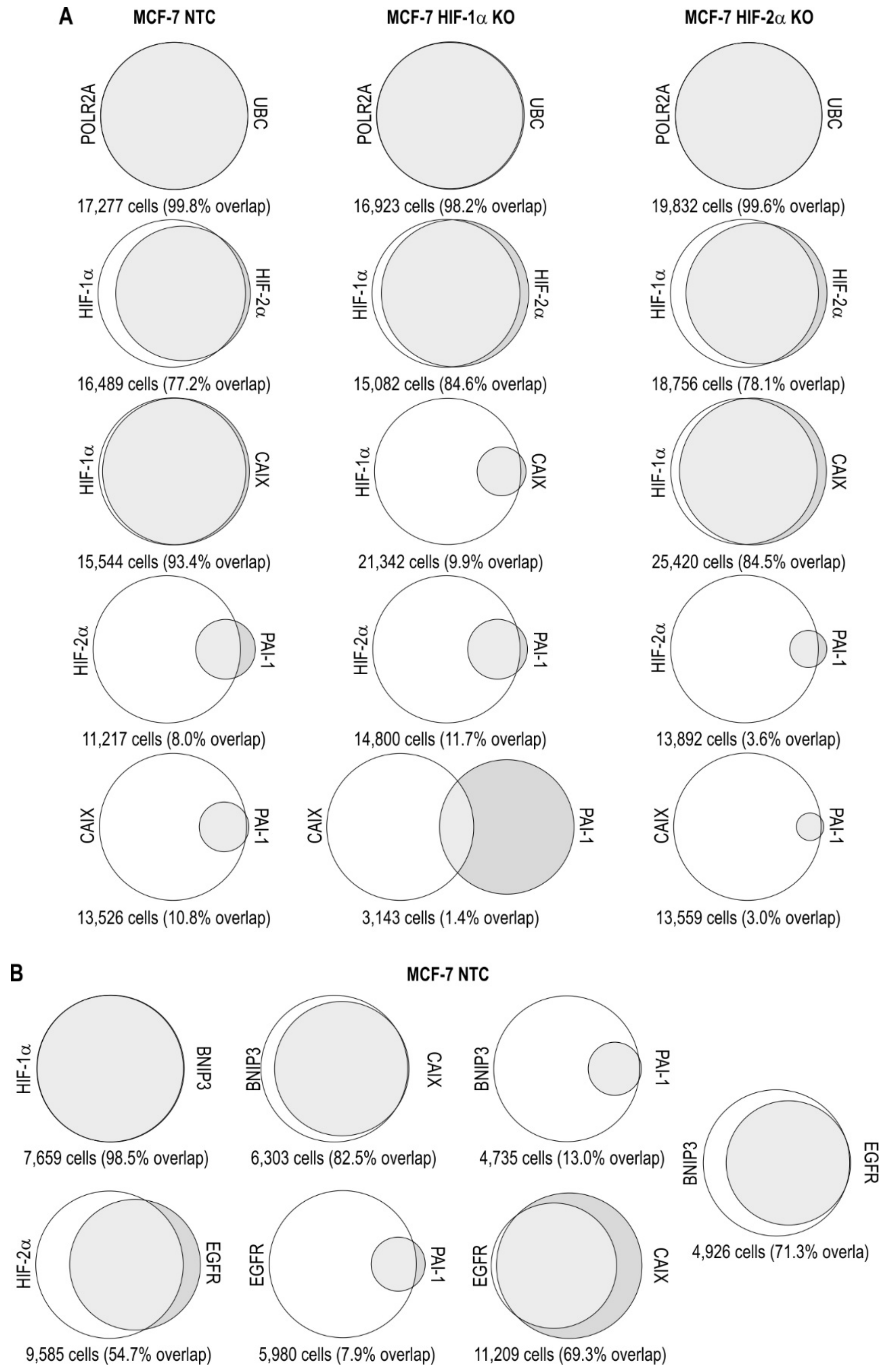

Supplementary Fig. S7. mRNA coexpression analyses. (**A**) MCF-7 NTC clone A4 and HIF-1α KO clone A3 or HIF-2α KO clone 2C4 cells were exposed to hypoxia for 48 hours, and coexpression was analyzed via multiplex mRNA-FISH. The proportion of double-positive cells is indicated by Venn diagrams. Regardless of its extent, a cell was considered positive when there was an mRNA signal above the background of a bacterial dapB mRNA-FISH probe that served as a negative control. The total number of cells analyzed is indicated. (**B**) Two additional HIFα isoform-specific target genes were analyzed in MCF-7 NTC cells as outlined in **A.**

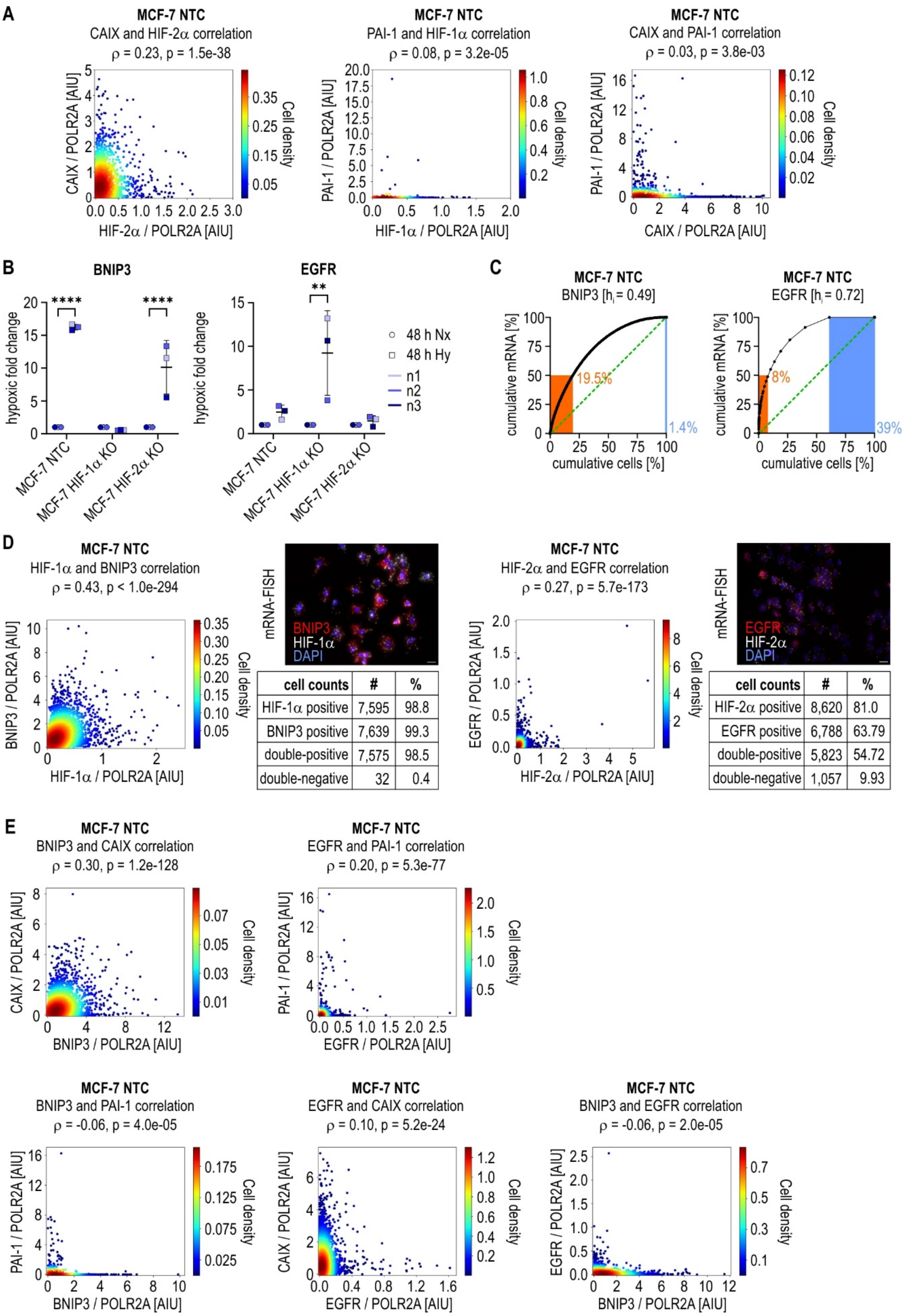

Supplementary Fig. S8. mRNA sc-correlation analyses. (**A**) MCF-7 NTC clone A4 and HIF-1α KO clone A3 or HIF-2α KO clone 2C4 cells were exposed to hypoxia for 48 hours, and the coexpression of the indicated genes was analyzed via multiplex mRNA-FISH. The fluorescence intensities of the mRNAs of interest were normalized to the average cellular intensity over all cells of POLR2A probes labeled with the same fluorophore. Data are shown in scatter plots (assembly of 3 independent

experiments; ρ, Spearman's correlation coefficient; AIU, arbitrary intensity unit). (**B** - **E**) Two additional HIFα isoform-specific target genes were analyzed in MCF-7 cells treated as outlined in **A**. (**B**) Transcript levels were quantified via RT-qPCR, divided by ribosomal protein L28 mRNA levels, and normalized to the corresponding normoxic controls. Two-way ANOVA followed by Bonferroni's multiple comparisons test was used to statistically evaluate differences compared with the normoxic controls. **p<0.01; ****p<0.0001. (**C**) sc-Heterogeneity indices (h_i) are shown in representative histogram charts. (**D**) HIF-1α/BNIP3 and HIF-2α/EGFR correlation analyses were performed as outlined in **A**. Representative fluorescence microscopy images are provided in the upper panels (scale bars = 20 μm). The tables in the lower panels list the number (#) and percentage (%) of cells with the indicated mRNAs over or below the detection limit. (**E**) Correlation analyses among the indicated HIF target genes.

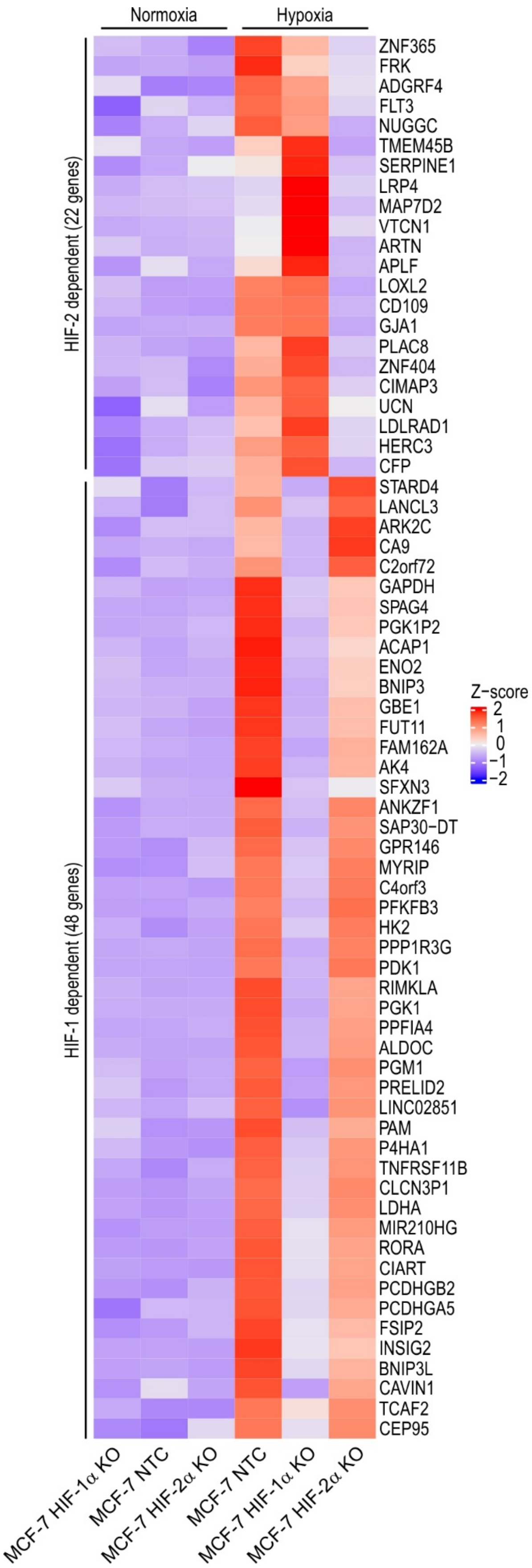

Supplementary Fig. S9. HIF target genes used for supervised UMAP analysis. MCF-7 NTC clone A4 and HIF-1α KO clone A3 or HIF-2α KO clone 2C4 cells were exposed to hypoxia for 48 hours, and the expression of the indicated genes was analyzed via scRNAseq. The heat map was generated based on a minimal hypoxic induction of log2(fold change) > 1.5 and a minimal expression of > 50 transcripts per kilobase million (TPM), and included 70 HIF isoform-specific target genes that were not induced anymore in either the HIF-1α or HIF-2α knockout cells.

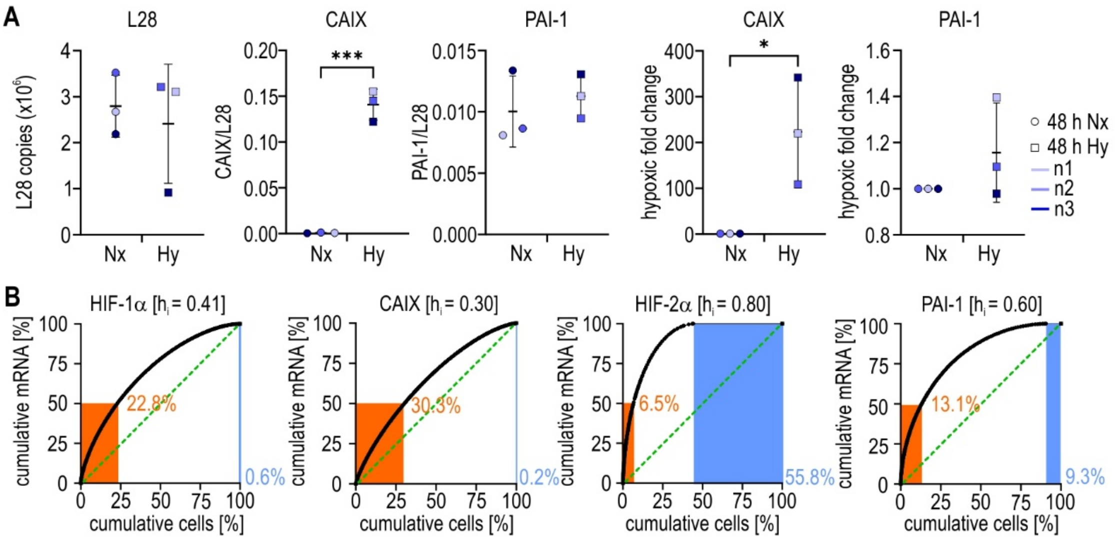

C

corrected to POLR2A average intensity	U2OS (n = 3)			
	HIF-1α	CAIX	HIF-2α	PAI-1
h_i	0.43 ± 0.03	0.32 ± 0.02	0.79 ± 0.04	0.63 ± 0.06
% contributing to 50%	22.1 ± 1.5	29.1 ± 1.6	7.2 ± 1.5	11.1 ± 3.9
% not contributing	2.2 ± 2.2	0.1 ± 0.1	57.6 ± 9.2	7.2 ± 2

D

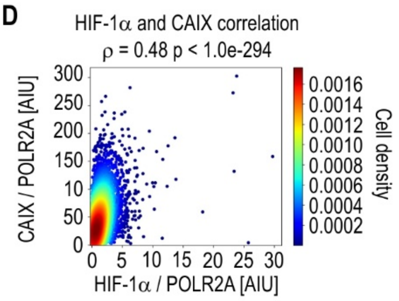

cell counts	#	%
HIF-1α positive	13,989	98.1
CAIX positive	14,246	99.9
double-positive	13,977	98.0
double-negative	0	0

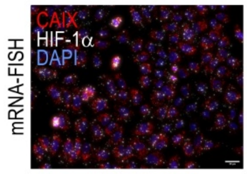

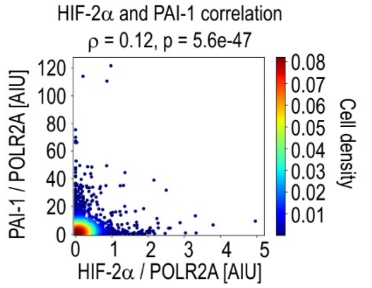

cell counts	#	%
HIF-2α positive	9,163	53.7
PAI-1 positive	16,039	93.9
double-positive	5,830	34.1
double-negative	701	4.1

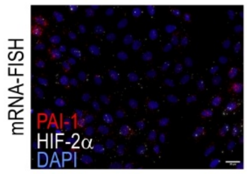

Supplementary Fig. S10. sc-Heterogeneity of the HIF response in U2OS cells. U2OS cells were exposed to normoxia (Nx) or hypoxia (Hy) for 48 hours. (**A**) Batch transcript levels were quantified via RT-qPCR, divided by the corresponding levels of the ribosomal protein L28 mRNA, and normalized to the normoxic controls. The mean values ± S.D. of n=3 independent experiments are shown. Unpaired t-test was used to statistically evaluate differences compared with the normoxic controls. *p<0.05; ***p<0.001. (**B**, **C**) sc-Transcript levels were analyzed by mRNA-FISH, and the data are displayed in representative histogram charts as outlined in Fig. 1. (**B**) and in a table containing assembled data (**C**). (**D**) Scatter plots of the sc-correlation analyses performed as outlined in Fig. 3. Exemplary fluorescence microscopy images are shown (scale bars = 20 µm), and the number (#) and percentage (%) of cells with the indicated mRNAs over or below the detection limit are listed.

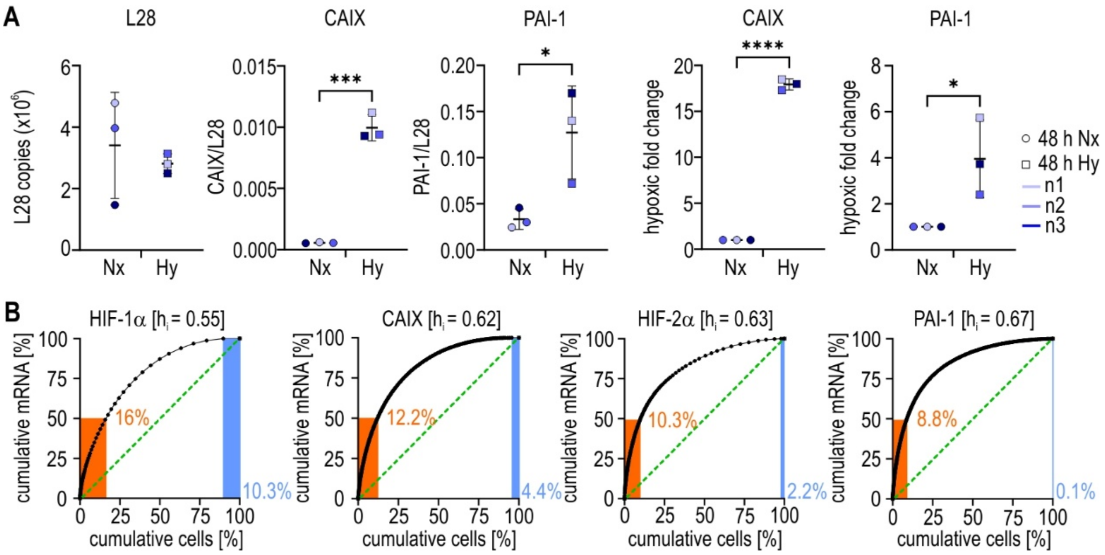

C

corrected to POLR2A average intensity	MDA-MB-231 (n = 3)			
	HIF-1α	CAIX	HIF-2α	PAI-1
h_i	0.58 ± 0.09	0.64 ± 0.1	0.64 ± 0.1	0.66 ± 0.02
% contributing to 50%	14.8 ± 3.6	11.4 ± 4	9.9 ± 4.2	9.7 ± 2
% not contributing	15.3 ± 9.4	6.4 ± 5.1	4.5 ± 4.5	0.1 ± 0.1

D

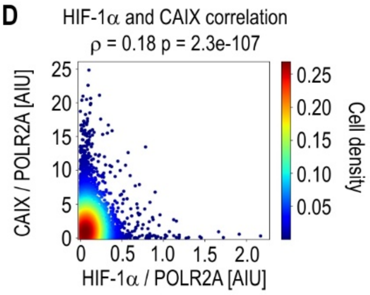

cell counts	#	%
HIF-1α positive	12,617	89.3
CAIX positive	13,334	94.4
double-positive	11,996	84.9
double-negative	175	1.2

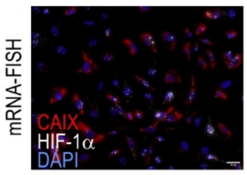

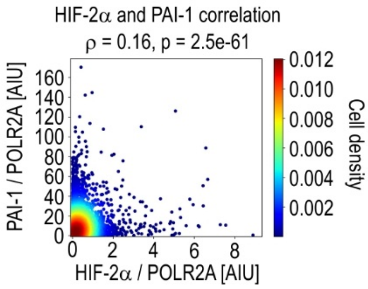

cell counts	#	%
HIF-2α positive	10,940	97.63
PAI-1 positive	11,197	99.92
double-positive	10,934	97.57
double-negative	3	0.03

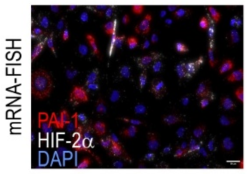

Supplementary Fig. S11. sc-Heterogeneity of the HIF response in MDA-MB-231 cells. MDA-MB-231 cells were exposed to normoxia (Nx) or hypoxia (Hy) for 48 hours. (**A**) Batch transcript levels were quantified via RT-qPCR, divided by the corresponding levels of the ribosomal protein L28 mRNA, and normalized to the normoxic controls. The mean values ± S.D. of n=3 independent experiments are shown. Unpaired t-test was used to statistically evaluate differences compared with the normoxic controls. *p<0.05; ***p<0.001; ****p<0.0001. (**B**, **C**) sc-Transcript levels were analyzed by mRNA-FISH, and the data are displayed in representative histogram charts as outlined in Fig. 1. (**B**) and in a table containing assembled data (**C**). (**D**) Scatter plots of the sc-correlation analyses performed as in Fig. 3. Exemplary fluorescence microscopy images are shown (scale bars = 20 µm), and the number (#) and percentage (%) of cells with the indicated mRNAs over or below the detection limit are listed.

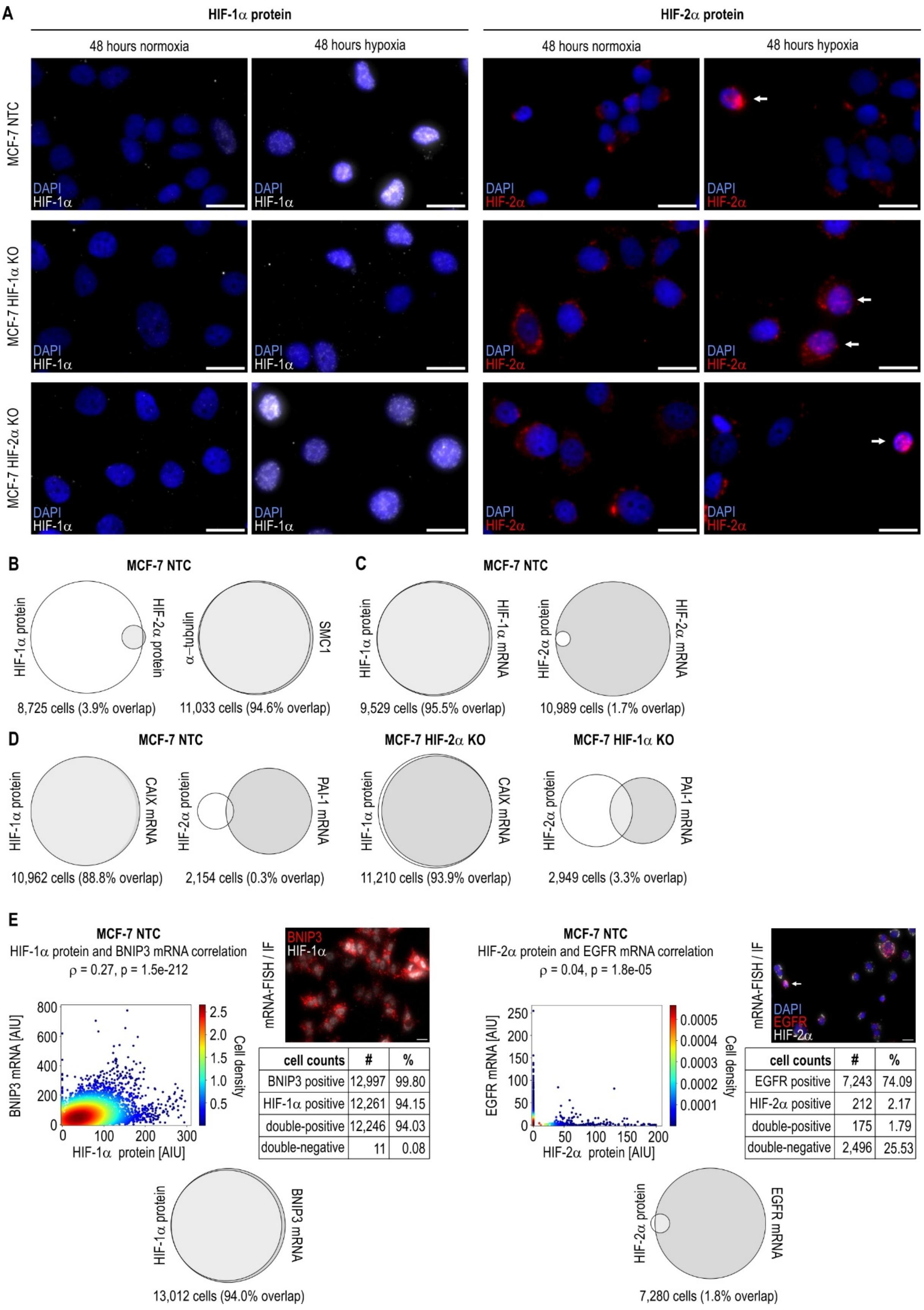

Supplementary Fig. S12. HIF-1α and HIF-2α protein sc-heterogeneity. (**A**) MCF-7 NTC clone A4 and HIF-1α KO clone A3 or HIF-2α KO clone 2C4 cells were exposed to normoxia or hypoxia for 48 hours, fixed and analyzed by immunofluorescence. (**B** - **D**) Venn diagrams of the indicated hypoxic cell lines analyzed by sequential immunofluorescence (**B**) or mRNA-FISH followed by

immunofluorescence (**C**, **D**). (**E**) MCF-7 NTC cells were treated as above, and the raw data are shown in scatter plots. Exemplary fluorescence microscopy images, tables with the number (#) and percentage (%) of cells with the indicated mRNAs over or below the detection limit, and Venn diagrams are included. (**A**, **E**) Arrows indicate cells positive for nuclear HIF-2α; scale bars = 20 μm.
